# Preparation and Calibration of Carrier-Free ^209^Po Solution Standards

**DOI:** 10.6028/jres.100.002

**Published:** 1995

**Authors:** R. Collé, Zhichao Lin, F. J. Schima, P. A. Hodge, J. W. L. Thomas, J. M. R. Hutchinson, B. M. Coursey

**Affiliations:** National Institute of Standards and Technology, Gaithersburg, MD 20899-0001

**Keywords:** alpha counting, lead-205, liquid scintillation (LS), measurements, polonium-209, radioactivity, standards

## Abstract

Carrier-free ^209^Po solution standards have been prepared and calibrated. The standards, which will be disseminated by the National Institute of Standards and Technology as Standard Reference Material SRM 4326, consist of (5.1597 ±0.0024) g of a solution of polonium in nominal 2 mol · L^−1^ hydrochloric acid (having a solution density of (1.031±0.004) g · mL^−1^ at 22 °C) that is contained in 5 mL flame-sealed borosilicate glass ampoules, and are certified to contain a ^209^Po alpha-particle emission rate concentration of (85.42±0.29) s^−1^ · g^−1^ (corresponding to a ^209^Po activity concentration of (85.83 ±0.30) Bq · g^−1^) as of the reference time of 1200 EST 15 March 1994. The calibration was based on 4πα liquid scintillation (LS) measurements with two different LS counting systems and under wide variations in measurement and sample conditions. Confirmatory measurements by 2πα gas-flow proportional counting were also performed. The only known radionuclidic impurity, based on α- and photon-emission spectrometry, is a trace quantity of ^208^Po. The ^208^Po to ^209^Po impurity ratio as of the reference time was 0.00124 ±0.00020. All of the above cited uncertainty intervals correspond to a combined standard uncertainty multiplied by a coverage factor of *k* = 2. Although ^209^Po is nearly a pure α emitter with only a weak electron capture branch to ^209^Bi, LS measurements of the ^209^Po a decay are confounded by an a transition to a 2.3 keV (***J***^π^= 1/2^−^) level in ^205^Pb which was previously unknown to be a delayed isomeric state.

## 1. Introductory Notes

Solution standards of polonium isotopes have been and are important in a variety of applied measurement disciplines. At present, they are primarily of interest because of their use as calibration standards for alpha-emission rate measurements, and as low-level tracers and separation yield monitors in radiochemical procedures that are employed with environmental and geophysical samples.

For many years, preceding the past one or two decades, ^210^Po was unquestionably the polonium isotope most widely used in nuclear physics and radiochemistry research [1]. The reasons for this past popularity are evident: it could be prepared relatively easily and with virtually no radionuclidic impurities by (d,n) reactions on natural monoisotopic ^209^Bi, although historically it most often has been obtained by extraction from very aged natural samples containing progenitors in the primordial ^238^U series; its half-life (138 d) is neither inconveniently too short nor too long for use in many physical and chemical studies; it has no intervening radioactive daughter products, decaying directly to stable ^206^Pb; and its decay is by 100 % monoenergetic alpha emission. Over the years, however, its popularity has somewhat waned because of the advantages inherent in having longer-lived standards for use within applied-measurement laboratories.

More recently, in 1984, the National Bureau of Standards (NBS), now NIST, prepared, calibrated and disseminated carrier-free ^208^Po solution standards. These longer-lived ^208^Po standards (2.9 yr half-life) were issued as NIST Standard Reference Material SRM 4327. They consisted of approximately 1.1 g of 1 mol · L^−1^ hydrochloric acid (HCl) contained in 2 mL flame-sealed borosilicate-glass ampoules, and were certified to contain a ^208^Po activity concentration of (76.6 ±1.1) Bq · g^−1^ on 20 June, 1984 [2]. The above cited uncertainty interval corresponds to a combined standard uncertainty multiplied by a coverage factor of *k* =3. The solutions contained a small quantity of contaminant ^209^Po with a ^209^Po to ^208^Po impurity ratio of 0.0065 ± 0.0026 at the reference time. The impurity ratio, of course, worsened with increasing age of the standard. These SRMs had a great demand from laboratories throughout the world and the initial supply of several hundred sources was exhausted within a few years.

Since the initial issuance of the SRM 4327 ^208^Po standards, considerable interest was expressed for having available similar standards of the even longer-lived 102 yr ^209^Po. In addition to the longer half-life, standards of the ^209^Po isotope also have the advantage of being capable of being prepared in a very pure state by ^209^Bi(p,n) reactions. Furthermore, the impurity ratios for any slight traces of the shorter-lived ^208^Po or ^210^Po impurities will of necessity improve with time.

The present work to prepare and calibrate a new series of ^209^Po solution standards follows a preceding companion study by Collé [3] in which he investigated the long-term stability of very dilute, aged solutions of polonium. Prior to this earlier study, the integrity of polonium solutions stored over a decade or so was largely unknown. Considering the complex and often-perplexing chemistry of polonium [4–6], the previous study was considered to be advisable prior to issuing any further polonium standards, such as that for ^209^Po described herein.

## 2. Methods, Results, and Discussions

### 2.1 Experimental Overview and Methodology

#### 2.1.1 Experimental Design

**Fig. 1:**
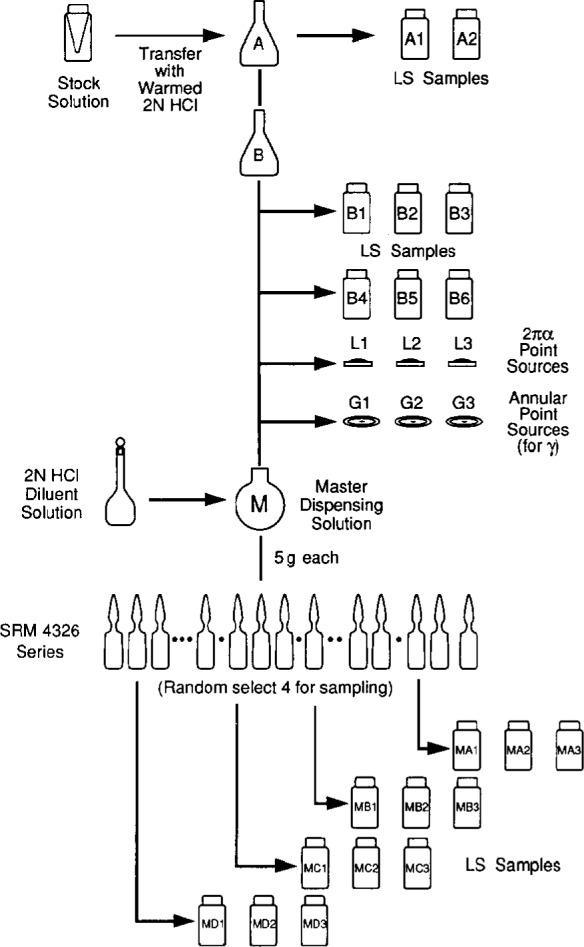
Schema for preparation of the ^209^Po standards and counting sources used for the calibration.

The initial experimental design for the preparation and calibration of the standards was found to require considerable subsequent modification. The original design is outlined in the schema of Fig. 1. This design was followed, but, because of some difficulties and inexplicable early findings in the liquid scintillation (LS) measurements, a number of additional independent LS calibrations were also performed.

As indicated in Fig. 1, the ^209^Po stock solution was transferred to a larger vessel (solution A) from which two LS samples were prepared. These solution A samples were intended to be used to obtain an approximate assay of the ^209^Po content prior to proceeding with the remaining scheme. Based on the findings of this assay, solution A could then be appropriately diluted to the starting point of solution B. Four sets of counting sources that were to be used for the primary calibration were gravimetrically prepared from solution B. The sources consisted of two sets of LS samples with two different quench conditions; three evaporated solid sources for use in α-emission impurity analyses and for confirmatory measurements by 2πα counting; and three evaporated solid sources for use in photonemission impurity analyses. A master dispensing solution (M) was also prepared by a careful gravimetric dilution of solution B. Approximately 200 ampoules of the ^209^Po standard were prepared from the master solution M. Four randomly selected ampoules were then used to prepare 4 sets of LS samples to estimate between-ampoule variability.

It was envisaged that the activity concentration of the ^209^Po in the ampoule standards would be obtained from 4πα LS measurements on the solution B samples after applying the known gravimetric dilution factor to the assay results. Samples B1 through B3 were minimally quenched, and samples B4 through B6 were intended to be matched to the quench conditions of the M samples (see Fig. 1). Comparison of the LS results for the matched B and M samples would therefore verify the dilution factor. Confirmatory measurements were also performed by 2πα gas-flow proportional counting of the evaporated solid sources L1 through L3. From the onset, it was considered that these solid source measurements would at best only serve as less accurate, but independent confirmations of the LS results. In contradistinction, the 1984 calibration of the previous ^208^Po standards [2] was based on defined-geometry α-particle scintillation counting of evaporated sources, and used LS counting as an independent confirmatory measurement method. This difference in the respective calibration approaches reflects, in part, the improvements in conventional LS standardization techniques over the past decade.

#### 2.1.2 Absolute Standardization by 4πα LS Counting and Problems Encountered with ^209^Po

**Fig. 2:**
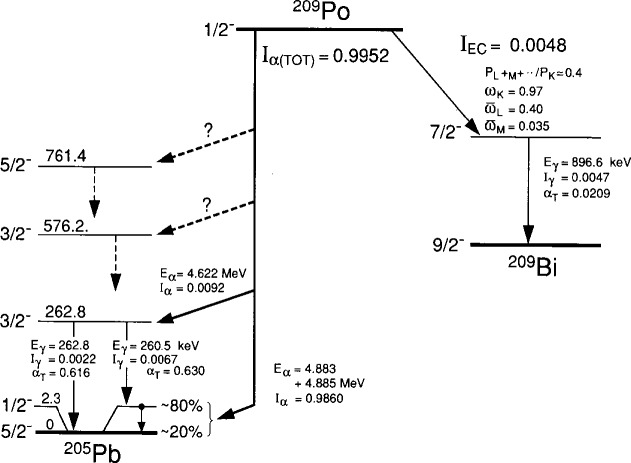
Partial decay scheme for the ^209^Po alpha and electron capture branch decays.

Absolute standardizations of pure α-emitting radionuclides by 4πα LS measurements are relatively straightforward.^1^ It was assumed, mistakenly, that this would be the case for this calibration. The LS detection efficiency for a particles is virtually 100 % and independent of reasonable sample quenching conditions. Therefore, it was assumed that a calibration could be obtained by merely measuring and integrating the full-energy LS spectra for the ^209^Po samples with appropriate corrections for background subtractions, for extrapolations of the spectra to zero energy, for the presence of any impurities, and for decay of the ^209^Po and any known impurities. The corrected LS count rate as a function of sample mass can also be extrapolated to zero sample mass (as a kind of quench correction), but this is seldom necessary with a emitters since the sample mass dependency is usually found to be completely invariant of quench conditions. Lastly, it was recognized that the LS calibration would require a correction for the small (*I*_EC_ = 0.0048 ± 0.0004) electron capture branch decay of ^209^Po (refer to Fig. 2).

The results of LS measurements based on the above described protocol evidenced a number of anomalies.

First, all of the LS samples were measured with two different LS counting systems, and the two sets of corrected results exhibited substantial systematic differences. Depending on the specific sample in question, the differences in LS response between the two LS instruments ranged from about 0.5 % to several percent. This result would suggest a difference in discrimination level or detection threshold for the two LS systems. Yet, this should not have been important for a nearly pure α emitter like ^209^Po. Such an instrument-dependent discrepancy had not been observed previously with other radionuclides, including β^−^ emitters like ^36^C1 where the effect could be expected to be more pronounced [10].

Second, the magnitude of the differences between the two instruments for a given sample appeared to be temporally dependent – i.e., dependent on the time of sample measurement. This, of course, suggested the possibility of the detection efficiency (and or quenching) changing with time. Again, this should not have been important for ^209^Po with an expected nearly quench-independent LS response.

Third, early LS samples prepared from solutions A and B exhibited very rapid changes in instrument quench indicating parameters. This too should not have affected the LS a detection efficiency.

Fourth, the dilution factors obtained from comparison of the LS results with the solution B and M samples (using only the results from a single instrument) were not in as good agreement with the gravimetrically-determined dilution factor as that expected. The LS-determined dilution factors (for either instrument) differed from the gravimetrically-determined one by a few tenths of a percent. The observed range of differences was of course due to the temporal variations noted above. Typically, for a single dilution, such dilution factor verifications differ by less than a few hundredths of a percent.

Fifth, and lastly, the LS spectra contained some structural details that were not understood or expected. The magnitude of some of the observed differences and effects could not be accounted for by any known non-α decay mode in ^209^Po, such as that from electron-capture branching.

Considering that these seeming measurement discrepancies were not experienced in many previous LS standardizations by this laboratory, the causative culprit was initially suspected to be either some peculiarity of polonium chemistry, some type of LS cocktail instability, or some combination of the two.

In an attempt to understand and reconcile the above observed discrepancies, a considerable number of additional LS samples were prepared for further investigations. In large measure, this further work involved a systematic study of the LS responses and spectral details as a function of different sample compositions and quenching conditions. The initial phases of the work was conducted in the period of March through June 1993 followed by an eight-month abeyance. Further systematic studies were continued and finally concluded in the period between February and April 1994.

#### 2.1.3 ^209^Po Decay Considerations

The cause of the initially discrepant LS results was finally understood during the 1994 period. It was deduced that they were not measurement discrepancies *per se*, but rather were a result of the initially chosen analysis method which was inappropriate when applied to the measurement of ^209^Po. The problem arose from an inherent, but unknown aspect of ^209^Po decay: a sufficiently long, delayed isomeric state in ^205^Pb. A partial decay scheme, based on the recent critical evaluation by Martin [11], is shown in Fig. 2. As indicated, the relative α branching to the two lowest energy levels in ^205^Pb is largely unknown. Neither of the two most complete studies of ^209^Po α decay [12,13] could resolve differences in an a transition to the ^205^Pb ground state (***J***^π^ = 5/2^−^) or to the 2.3 keV (*J*^π^ = 1/2^−^) first excited state. The energy of the principal a branch (*E*_α_ = 4.883 MeV) measured in these studies is assumed to correspond to the branch to the 2.3 keV level. The α transition to the ground state is thus 2 keV larger, i.e., *E*_α_ = 4.885 MeV. The measured intensity for this combined a transition (*I*_α_ = 0.9860 ± 0.0008) is that for populating both levels. The assumed relative populations of ≈20 % to the ground state and ≈80 % to the 2.3 keV level are based on estimates by Martin [11] using the systematics of α-decay hinderance factors. Irrespective of the absolute α transition intensity to the 2.3 keV level, it seems clear that it is probably populated to a substantial extent. Furthermore, it is possible that the decay of this state is delayed and not in a prompt coincidence with the α feeding of it. The resulting 1/2^−^→5/2^−^ transition is also likely to be very highly converted with the emission of 2.3 keV conversion electrons (ce). If the lifetime of this 2.3 keV state is comparable or longer lived than the pulse resolving time of the LS counting systems, than some fraction of these ce (and attendant 2.3 keV *γ* rays, if any) would be detected independent of the feeding α-decay branch. The LS detection efficiency for the ce would also be dependent on sample quenching conditions and on the specific discrimination levels or detection thresholds for the LS instruments.

Detailed examinations of the LS spectra as a function of quench conditions verified these conjectures. The 2.3 keV ce transition was identified in spectra from both LS counters. A distinct low-energy peak centered at about 3 keV was observed. Its large relative magnitude, ranging from just less than 1 % to nearly 6 % in various spectra, excluded the possibility of attributing the peak to other non-α decay modes, e.g., to Auger electrons from the weak electron capture decay branch. Its magnitude was also quench dependent, and was invariably larger in the instrument having a shorter coincident pulse resolving time. When this ce component was subtracted from the total LS response and spectra, one could obtain a virtually quench-independent a emission rate. Although the total LS detection efficiencies (which included the 2.3 keV component) for the two LS counting systems differed substantially, the subtracted α emission rates obtained from both instruments where in excellent agreement and invariant of quench conditions.

Based on these findings, all of the original 1993 results were also successfully re-analyzed and found to be in nearly perfect agreement with the 1994 results. Hence, this standardization of ^209^Po was based on essentially two completely independent determinations that were separated by nearly a year in time, each of which was also based on the use of two different and independent LS counting systems. The standardization may be considered to be somewhat over-determined! It ultimately involved nearly 500 independent LS measurements with each instrument using over 40 different LS samples (covering a diverse range of sample compositions and quenching conditions) which were obtained from 9 different ^209^Po solutions having gravimetrically related concentrations.

#### 2.1.4 Measurement Uncertainty Treatments

The specification of measurement uncertainties and the uncertainty analysis procedures used throughout this paper follow the normal conventions of the NIST Radioactivity Group. These conventions are wholly compatible with those adopted by the principal international metrology standardization bodies [14,15]. All individual uncertainty components are expressed in terms of estimated (experimental) standard deviations (or standard deviations of the mean where appropriate) or quantities assumed to correspond to standard deviations irrespective of the method used to evaluate their magnitude. All of these component quantities are designated as “standard uncertainties.” A propagated uncertainty, termed a “combined standard uncertainty,” is expressed as what is assumed to be an estimated standard deviation which is equal to the positive square root of the total variance obtained by summing all variance (square of the standard uncertainty) and covariance components, however evaluated, using the law of propagation of uncertainty for the specific mathematical function given by the model of the measurement procedure [14]. By recently established NIST policy [15], the combined standard uncertainty is multiplied by a “coverage factor” of *k* = 2 to obtain an “expanded uncertainty” which is assumed to provide an uncertainty interval having a level of confidence of roughly 90 % to 95 %. For comparative purposes, it should be noted that previous SRM certificates issued by the NIST Radioactivity Group used comparably-based uncertainty coverage factors of *k* = 3. This former practice was historically rooted and was assumed to provide certified uncertainty intervals with somewhat higher confidence levels, approaching 95 % to 99 %.

Many of the component standard uncertainty estimates, such as those for gravimetric determinations, detection efficiencies, and various correction factor and extrapolation procedures, are based on canonical values or on approximations by established procedures which were in turn determined by diverse experimental verifications and inferences and which were established by this laboratory over many years of experience.

### 2.2 Solution, Counting Source and Standard Preparations

#### 2.2.1 ^209^Po Stock Solution

The ^209^Po stock solution used to prepare the standards has a somewhat uncertain history. It was obtained by NIST from Oak Ridge National Laboratory (ORNL) in May 1989; and, based on partial records, appears to have been originally produced and radiochemically separated by Mound Laboratory at least several years earlier [16]. The high degree of radionuclidic purity found in subsequent impurity evaluations suggests that it most probably was prepared by low-energy (p,n) reactions on natural monoisotopic ^209^Bi. The stock solution, contained and shipped in a 1 mL glass minivial, consisted of a nominal 100 kBq ^209^Po as polonium nitrate in about 0.6 mL of 5 mol · L^−1^ nitric acid (HNO_3_).

#### 2.2.2 Solutions A and B

The stock solution was transferred and diluted with about 8 g of warmed 2 mol · L^−1^ HCl using an aspirating plastic pycnometer for the transfer and subsequent rinsing of the minivial. The resulting solution (labelled A in Fig. 1) was contained in a 20 mL wide-mouth glass vessel. Two gravimetrically determined aliquants of the solution were used to prepare two LS samples (described below). Rudimentary LS assays of these samples indicated that most of the polonium was transferred; roughly 97 % of that said by ORNL to be contained in the minivial. If this had not been the case (as has sometimes been found in trying to remove polonium from glass vessels [3]), then more heroic efforts could easily have been made to recover the polonium remaining in the minivial and adding it to solution A. This fortunately was unnecessary in this particular transfer.

Solution A was subsequently kept warmed and unstoppered over a period of about 60 h in an attempt to remove or decrease the HNO_3_ concentration in the solution by evolving NO_2_. Warming of the solution was maintained with an infrared heat lamp at temperatures around 60 °C to 70 °C. Higher temperatures were not employed in fear that the polonium might be hydrolyzed or volatilized and thereby lost from solution [4–6]. The resulting solution was labelled B. The activity concentrations of solutions A and B should hence be equal except for possible small evaporation losses during the warming. Solution B was then used to prepare the four sets of counting sources that are noted in Fig. 1 along with a master dispensing solution M.

#### 2.2.3 Gravimetric Determinations

All of the solution aliquants used to prepare the counting sources and solution M were determined gravimetrically with an estimated uncertainty, corresponding to an assumed standard deviation, of ± 10 μg to ±30 μg. The mass determinations were made for the most part with single-pan suspension microbalances having internal balance reference weights. For sample aliquants ranging from 50 mg to 100 mg for the counting sources, the relative standard uncertainties in the sample mass measurements thus are about ±0.01 % to ±0.03 % in general, and about ±0.06 % in worst cases, after appropriate gravimetric measurement air-buoyancy corrections, and considering the internal-balance-weight uncertainties, and typical mass measurement precision. Subsequent LS samples prepared from sealed ampoules of master solution M used sample masses typically ranging from 0.1 g to 1 g and thus had relative standard uncertainties of roughly ±0.05 % in worst cases. For solution M which had an aliquant sample mass of greater than 7.5 g, the relative standard uncertainty in the sample mass is less than 0.001 percent. In all cases, aliquant sample masses were redundantly and independently determined from pycnometer-dispensed mass differences and from contained masses obtained from the differences between the final sample masses and initial tare masses of the containing vessels. Differences in the two mass determinations are typically less than 0.05 percent for sample masses greater than 100 mg. This type of sample mass verification was employed throughout the preparation of all samples given in this paper, and has mass uncertainties typical for the routine gravimetric procedures used in our laboratory. The uncertainty in the gravimetric dilution factor between solutions B and M is of course dominated by the uncertainty in the large HCl diluent mass which is in turn dominated by the uncertainties in the internal balance weights for the employed 2 kg single pan suspension balance, and has an estimated overall relative standard uncertainty of about ±0.05 %.

#### 2.2.4 Master Solution M and Ampoules of the ^209^Po Standard

In preparing solution M, solution B was diluted with 2 mol · L^−1^ HCl by a gravimetrically determined factor of *D* = 147.243 ±0.074 (about 7.5409 g of B in 1110.3 g of M).

The solution M diluent was a carefully prepared HCl solution having a nominal acid concentration of 2.0 mol · L^−1^ and density of (1.031 ±0.002) g · mL^−1^ at 22 °C. It was prepared from NIST high-purity, vacuum-distilled hydrochloric acid (nominal 9.8 mol · L^−1^) in doubly-distilled water. The uncertainty in the density is an estimated standard deviation based on independent gravimetric determinations of the total master solution mass and individually dispensed masses in known dispensing volumes. This identical HCl solution was also used in the transfer of the stock ^209^Po solution to solution A. It along with a blank doubly-distilled water solution was also used in preparing some of the subsequent LS samples. The blank water and HCl solutions were used to vary the HCl content in some of these LS counting sources, to vary or match sample compositions (and hence sample quenching) for the different LS sources, and to prepare matched blanks of nearly identical composition for background subtractions.

**Fig. 3:**
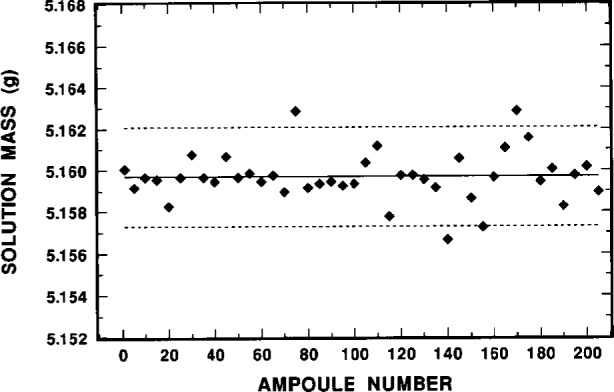
Dispensed solution masses of the ^209^Po standards as a function of ampoule number filling. The solid line is the mean of 42 determinations. The broken lines are upper and lower uncertainty limits for plus and minus two standard deviations about the mean.

To prepare the ^209^Po standard sources, approximately 5 mL of solution M was dispensed into each of 210 or so NIST 5 mL borosilicate-glass ampoules. Forty two, or about one out of every fifth ampoule that was filled, had been pre-weighed and then post-weighed to obtain an estimate of the dispensing precision and uncertainty in the contained solution mass. The ampoules were flame sealed after filling and weighing. The mean mass of the dispensed solution in the ampoules was (5.1597 ± 0.0012) g. The uncertainty given here is the standard deviation of the 42 individually weighed masses of solution from which the mean was calculated. It corresponds to a relative standard deviation of the mean of 0.0036 %. Figure 3 illustrates the dispensing precision as a function of ampoule filling. One may note that the mass variations appear to be somewhat larger with the later fillings (higher ampoule numbers) compared to that at the beginning (lower ampoule numbers). It is quite probable that this is not actually due to poorer dispensing precision into later ampoules, but rather is due to greater imprecision in the mass measurements at later times. One can speculate that this arose as the result of increasing imprecision in the balance readings introduced by fatigue of a laboratory worker who had to solely fill and seal 200^+^ ampoules, along with pre- and post-weighing a substantial fraction of them, all of which was performed over the course of 5 h.

#### 2.2.5 2πα- and Photon-Emission Point Sources

Three 2πα point sources (labelled L1 through L3 in Fig. 1) were prepared by depositing gravimetrically determined aliquants of solution B onto 2.5 cm diameter polished stainless steel (SS) disks. The deposits ranged from 39 mg to 51 mg of solution B, were dispersed and seeded with Ludox (E.I. DuPont de Nemours, Wilmington, DE),^2^ and then were evaporated to dryness without heating in ambient air. The acid content of solution B, however, seriously corroded and pitted the surfaces of the SS source mounts which was probably intensified by remaining traces of HNO_3_ in the solution. The sources were found to be unusable for quantitative a counting or a spectroscopy. It is also suspected that polonium may have been partially lost from the sources since the very low observed a emission rates (27 % to 36 % of that expected) seems too extreme to be accounted for solely by source self-absorption losses. Two additional sources (not shown in Fig. 1) were prepared at a later time and labelled LX1 and LX2. They consisted of 207 mg and 66 mg, respectively, of gravimetrically determined aliquants of solution M (obtained from a randomly selected ampoule of the standard solution) which were deposited onto 2.5 cm diameter platinum disks. The somewhat more massive solution deposits were used because of the lower activity concentration of solution M. The deposits covered a surface area of roughly 4.5 cm^2^ and 1.5 cm^2^ and were similarly evaporated to dryness in air. Sources LX1 and LX2 were used both for confirmatory measurements by gas-flow proportional counting and for spectroscopic analyses of α-emitting impurities.

Three point sources (labelled G1 through G3 in Fig. 1) for *γ*- and x-ray measurements were also prepared. They consisted of 32 mg to 82 mg of solution B deposited onto annular source mounts backed with ≈7 mg · cm^−2^ plastic tape (a glued polyester film). After the deposits air dried, the sources were covered and sealed with an identical layer of the tape.

#### 2.2.6 LS Counting Sources

The sample compositions for the LS counting sources are summarized in Table 1. Excepting a few samples that were prepared for special spectral investigations (and which will be noted later), the LS counting sources, in all cases, consisted of an appropriate aliquant of one of the ^209^Po solutions and approximately 10 mL or 14 mL of a prepared commercially-available LS cocktail mix contained within nominal 22 mL LS counting vials. The cocktail was “Ready Safe” (Beckman Instruments, Fullerton, CA), a prepared scintillation fluor in a polyarylalkane (i.e., an alkylated biphenyl) solvent having surfactant agents [17]. Some of the sources also contained respective proportions of a blank doubly-distilled water solution and the carefully prepared blank HCl solution. The water and HCl solution additions were done, as noted earlier, to vary the LS sample compositions to study sample quenching effects. In all cases, the sources were compared with matched blanks of nearly identical composition for background subtractions. The 1993 LS samples (A1 through N6 in Table 1) were prepared in high-density polyethylene LS vials, whereas the 1994 sources were contained in conventional glass LS vials. The change in vials was made to exclude the possibility that the initially inexplicable LS results were due to the use of plastic vials.

Table 1 summarizes the specific ^209^Po solution used to prepare the sources; the range for the mass *m*_s_ of the ^209^Po solution aliquant used in the source; the range for the total source mass *m*_tot_ (which is the sum of *m*_s_ and the masses of the scintillation cocktail, *m*_r_, and any blank water, m_w_, or blank HCl, *m*_a_, used in the source); the range for the aqueous phase content in the source, expressed as a percentage (*p*_aq_ = 100 · [m_s_ + m_w_ + *m*_a_]/*m*_tot_); and the range for the nominal normality of the acid content in the aqueous phase (*N*_aq_ = 2.0 [*m*_s_ + m_a_]/[*m_s_ + m*_w_*+m*_a_]). Although the values given in Table 1 are summarized in terms of approximate ranges, it should be recalled that each of the *m*_s_, *m*_v_, *m*_a_, and *m*_r_, masses in every sample was determined with an estimated relative standard uncertainty of ±0.05 %.

Samples A1 and A2 and B1 through B3 were prepared nearly identically with about *m*_s_≃52 mg to 65 mg of solution A or B in about *m*_r_≃10 g of cocktail. Corresponding matched blanks contained *m*_a_≃51 mg to 60 mg of the blank HCl solution in *m*_r_≃10 g of cocktail. Samples B4 through B6 were prepared with *m*_s_≃50 mg and *m*_w_=1 g in *m*_r_=10 g. The additional water in these samples would essentially match the total sample masses (or volumes) of the MA1 through MD3 sample series. Use of the blank water also more closely matched the quenching conditions in the two sample sets. Following an initial series of LS measurements on samples B4–B6, the samples were adjusted to the approximate *N*_aq_ = 2 mol · L^−1^ acid normality of the M samples. This was achieved by the addition of approximately 0.25 g to 0.3 g of the very pure NIST concentrated HCl. These adjusted samples are identified in Table 1 as samples B4x through B6x. The corresponding matched blanks were similarly adjusted. Four randomly selected sealed ampoules of the ^209^Po solution standard were used to prepare the 12 M series samples shown in Fig. 1. Each contained *m*_s_≃1 g in *m*_r_≃10 g. All further samples (not shown in Fig. 1) were also prepared using the sealed ampoules. Sample series N1 through N6, P1 through P5, Q1 through Q4, and Q5 through Q8 (along with all of their corresponding blanks) were prepared with varying quantities of *m*_s_, *m*_w_, *m*_a_, and *m*_r_ to achieve varying *p*_aq_ and *N*_aq_ sample compositions that could be used to systematically study cocktail stability, sample quenching, and spectral effects. Compositions of samples used in special spectral investigations are given later.

### 2.3 LS Measurement Systems

#### 2.3.1 Instruments

The two LS counting systems were: (1) a Beckman LS7800 model LS counter equipped with two Hamamasutu R331-05 photomultiplier tubes (PMT) operating in a coincidence mode, a logarithmic pulse amplifier coupled to an analog-to-digital converter (ADC) for spectral pulse-height analysis, and an external ^137^Cs source for Compton-edge (Horrocks number) quench monitoring [18]; and (2) a Packard Tri-Carb 2500TR LS analyzer employing two matched high performance PMT also operating in coincidence, but with linear amplification for ADC pulse-height spectral analysis, and an external low-energy γ-ray ^133^Ba source that is used to obtain an instrument-provided “transformed Spectral Index of the External Standard (*tSIE*)” quench indicating parameter [19].

Resolving times for the two instruments are slightly different [20,21]. Their relative magnitudes were particularly of interest in terms of interpreting the data in regard to the existence of the delayed isomeric state in ^205^Pb. The resolving times of the coincidence gates for simultaneous detection of singles PMT events were comparable: *τ*_c_ = 22 ns and *τ*_c_ = 18 ns in the Beckman and Packard instruments, respectively. Of greater interest, are the resolving times (i.e., equivalent deadtimes) for detection of coincident pulses. These were: *τ* ≃ 5 μs to 33 μs in the Beckman, and *τ* ≃ 12 μS in the Packard. The latter instrument has a fixed (i.e., “non-extending”) resolving deadtime. The *τ* ranges for the former result from the resolving time’s dependence on pulse height. The spectrometer would of course spend the vast majority of its time counting the principal ^209^Po α. Using the pulse heights for the dominant α peak (i.e., its channel number locations), the typical resolving times for the Beckman instrument were *τ* = (15±4) μS.

#### 2.3.2 Quench Indicating Parameters

The Horrocks number (*H^#^*) quench indicating parameter used with the Beckman system is based on the downward spectrum shift of the Compton edge of the external ^137^Cs *γ*-ray standard with increasing sample quenching. The parameter corresponds to the spectral channel number shift between the quenched sample and an unquenched blank sample. The channel number shift (*c*_2_*−c*_1_) is, in this case, equal to a logarithmic energy ratio log(*E*_2_/*E*_1_).

The Packard system’s quench indicating parameter *tSIE* is based on a proprietary mathematical transform of the energy distribution of the ^133^Ba generated Compton spectrum. The transform is said to be used to correct for spectral distortions arising, for example, from wall effects, volume variations, and color quenching. The parameter consists of a relative quenching scale in which unquenched samples correspond to *tSIE* = 1000.

**Fig. 4:**
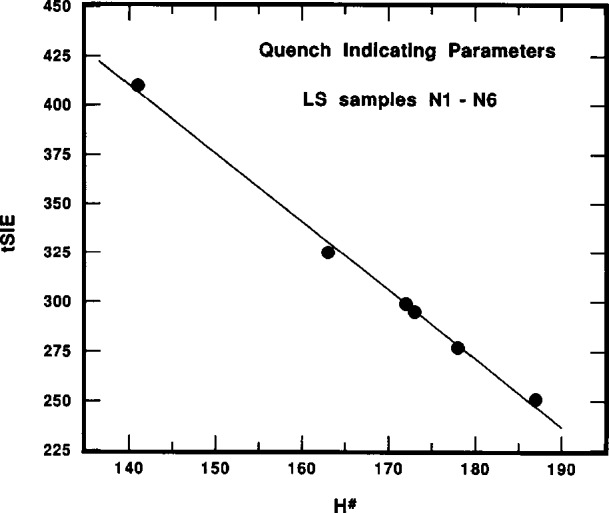
Correlation between the quench indicating parameters *H*^#^ and *tSIE* for LS samples Nl through N6.

**Fig. 5:**
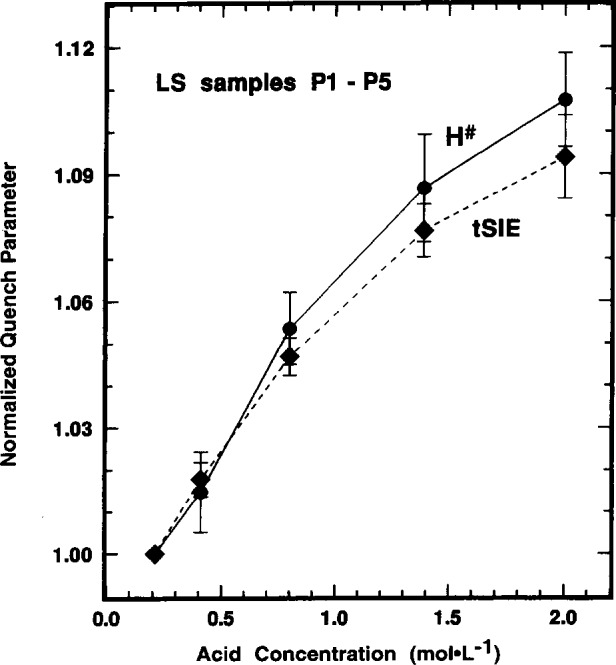
Quench indicating parameters as a function of the acid content *N*_aq_ in LS samples P1 through P5 normalized to the least quenched sample. Refer to text for normalizations.

The quench parameters *H^#^* and *tSIE* are inversely correlated and scale proportionally with variations in the LS sample composition that effect the chemical quenching. Figure 4, for example, illustrates the correlation between the two parameters for the N1 through N6 samples prepared and measured in 1993. The slope d(*tSIE*)/d(*H*^#^) corresponds to a decrease of about 3.5 units of *tSIE* for every unit of *H*^#^. A typical and representative illustration of the effect of LS sample composition changes is shown in Fig. 5. It shows the relative change in the two quench parameters as a function of the acid content *N*_aq_ in the P1 through P5 samples which were prepared and measured in 1994. The values given in Fig. 5 are ratios of *H*^#^/(93.65±0.69) and (439.8*±*1.2)/*tSIE* that consist of normalizations to the quench parameters for the least quenched P5 sample. The quench parameters similarly scale with other LS sample composition variables such as *m*_s_ or *p*_aq_ (see Table 1).

### 2.4 1993 4πα LS Measurement Results

#### 2.4.1 Analysis Procedures

The results given here are based on the 1994 re-evaluation of the originally collected 1993 data. This re-evaluation took account of the confounding by the 2.3 keV delayed isomeric state in ^205^Pb. Two corrected LS counting rate concentrations were evaluated. The first *R*_αe_ is that obtained from integrating the full-energy LS spectra, while the second *R*_α_ is that obtained after subtraction of the component responses from the 2.3 keV ^205^Pb transition. The integrated LS spectra energy regions, in terms of approximate beta energy, for the two are roughly from about Δ*E*_αe_ = 0 keV to 2000 keV for *R*_αe_, and from about Δ*E*_α_ ≃ 30 keV to 2000 keV for *R*_α_. The two counting rate concentrations are given by

$$\begin{array}{c} R_{\alpha e}=\frac{\left(C_{\alpha e}-C_{b\alpha e}\right)}{\left(e^{-\lambda_{209}T}+Ie^{-\lambda_{208}T}\right)tm}\\ R_{\alpha }=\frac{\left(C_{\alpha }-C_{b\alpha }\right)}{\left(e^{-\lambda_{209}T}+Ie^{-\lambda_{208}T}\right)tm} \end{array}$$

where

*C*_αe_: gross integral counts in the region Δ*E*_αe_ obtained from a spectrum of a given LS source at measurement time *T*;
*C*_bαe_: the corresponding gross integral counts in region Δ*E*_αe_ for background obtained from a spectrum of a matched blank LS sample;
*C*_α_: gross integral counts in the region Δ*E*_α_ of the source spectrum;
*C*_bα_: the corresponding gross integral counts in the region Δ*E*_α_ for the background spectrum;
*t*: counting (live) time interval for measurement of both the source and matched blank;
*m*: mass of the ^209^Po solution used in the LS source;
λ_209_: ^209^Po decay constant;
λ_208_: ^208^Po decay constant;
*T*: the decay time interval from the midpoint of the measurement time interval to a common reference time;
*I*: ^208^Po to ^209^Po impurity ratio in terms of their respective a emission rates at the reference time.

The difference *R*_e_ = (*R*_αe_*−R*_α_) is, of course the approximate LS counting rate concentration contribution due to the ^205^Pb 2.3 keV transition (presumed to be due predominantly to ce).

Two very small corrections, both less than 0.1 % and of opposing magnitude, were later applied to *R*_α_ to obtain the ^209^Po solution standard α-particle emission rate concentration *A*_α_. The first correction factor *k_ZE_* consisted of an extrapolation of *R*_α_ to zero energy by extrapolating *R*_α_ under the *R*_α_ portion of the spectra. The second correction factor *k*_EC_ was to remove LS response contributions arising from the EC branch of ^209^Po decay from *R*_α_.

The lower limits of the energy regions Δ*E*_α_ used to evaluate the 1993 data were slightly different for the two LS systems: for the Beckman instrument, Δ*E*_α_⪞30 keV (A and B sample series) and Δ*E*_α_⪞45 keV (M and N sample series); for the Packard, Δ*E*_α_⪞19 keV (all sample series). Evaluation of the later 1994 LS data also used slightly different integrating regions (see Sec. 2.5). The conversions of spectral channel numbers to a beta energy scale are, at best, only very approximate. First, one must recognize that the light energy transfer mechanisms in the scintillator/solvent mixtures are very non-linear at low energies. Second, the channel number to energy conversions are dependent on sample quenching. Third, and lastly, it is difficult to directly compare the spectra from the two LS systems since the Beckman instrument with its logarithmic amplification gives ADC channel numbers corresponding to a logarithmic energy scale while the conversion of channel number to energy is nominally linear with the Packard.

**Fig. 6:**
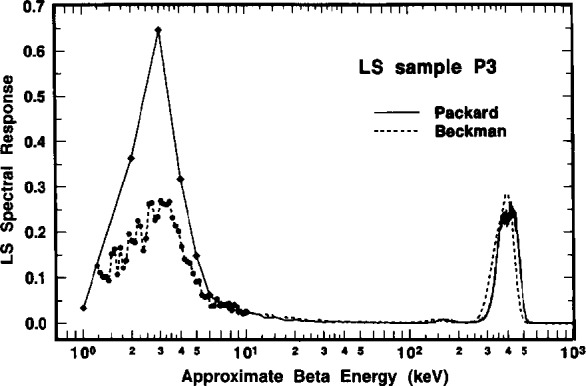
Comparison of the ^209^Po LS spectra obtained with the Beckman and Packard instruments.

A comparison of the ^209^Po LS spectra obtained with the two instruments is shown in Fig. 6. The spectra were obtained in 1994 with LS sample P3. The LS response from the dominant 4.88 MeV α of ^209^Po appears as a peak at around roughly 300 to 500 keV in the corresponding beta energy scale. This results from a particles (with their relatively short ranges) having scintillation yields that are typically a factor of 8 to 12 lower than that from beta emitters. The LS response from the ^205^Pb conversion electron, centered at approximately 3 keV, is readily apparent in both spectra. The relative magnitudes of the conversion electron (*R*_e_) and alpha peaks (*R*_α_) shown in the spectra may deceive on first appearance. One should note that the ordinate axis is in units of counts per second *per keV*, and that the abscissa is a logarithmic energy scale. The ratios of *R*_e_/*R*_α_ in the two spectra of Fig. 6 are actually 0.026 and 0.044 for the Beckman and Packard instruments, respectively. The large difference in the *R*_e_/*R*_α_ values may be attributed to differences in detection and resolution of the ^205^Pb conversion electron (ce) by the two instruments. One may also note that in the region below 10 keV, the spectrum obtained with the Packard instrument has only 9 or 10 channels of data while that from the Beckman instrument has over 50. Values of *R*_α_ for the two spectra shown in Fig. 6 are in better than 0.2 % agreement. In the region above about 550 keV, both spectra have net counting rates after background subtraction of virtually zero. The spectra in the region from about 30 keV to 100 keV are very flat and have net counting rates that are also very small which permits a relatively easy extrapolation of *R*_α_ below the *R*_e_ peak. Additional spectral analysis considerations are treated below (Sec. 2.6).

The 1993 LS measurements were based on averages of 5 to 10 replicate measurements of each sample interspersed with background measurements of blank samples. Each measurement ranged from 30 min to 120 min in duration. Because the initial results were not understood many of the samples were remeasured from 2 to 5 more times (with each subsequent sequence consisting of 5 to 10 measurements) over periods of nearly 100 d. The averaged results for *R*_αe_ and R_α_ were decay corrected to the common reference time of 1200 EST 15 March 1994 for direct comparison with the 1994 LS results. Values of *T* used for the decay corrections therefore often exceeded a year. The value of *I* = 0.00124 used for the impurity correction was independently obtained from α spectrometry (see Sec. 2.8).

#### 2.4.2 A and B Series Samples

**Fig. 7:**
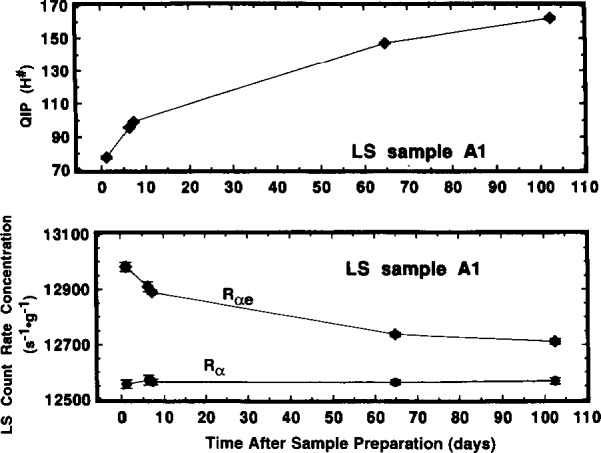
Quench parameter *H^#^* (upper trace) and corrected LS counting rate concentrations (lower trace) for LS sample A1 as a function of the time after sample preparation as obtained from measurements with the Beckman instrument. The upper curve in the lower trace corresponds to the counting rate concentrations *R*_αe_ obtained from integrating the full-energy LS spectra; the lower curve is for *R*_α_ and is that obtained after subtraction of the component LS responses from the 2.3 keV ^205^Pb transition.

All of the A and B series LS samples exhibited large and not very systematic variations in both quench indicating parameters and in values of *R*_αe_. The measurement results for sample A1 as a function of measurement time, shown in Fig. 7, are typical. Each plotted value in Fig. 7 is based on the mean of 5 to 10 measurements with the Beckman instrument. Over a period of approximately 100 d, the quench parameter *H^#^* varied from about 75 to over 160, with the largest rate of change occurring in the first 10 or so days. The Packard’s corresponding *tSIE* for sample A1, over a shorter 55 d period, ranged from 386 to 261. For 5 to 10 replicate measurements, these quench parameters *H^#^* and *tSIE* were typically determined with a precision having a relative standard deviation of the mean (*s*_m_) of 0.1 % to 0.3 %. Along with the change in *H^#^*, the mean values of *R*_αe_ over the same time ≃100 d interval decreased by over 2 %. The counting results for every A and B series sample measured with either instrument exhibited similar changes but of slightly differing magnitudes.

Later LS samples prepared from the standard solution ampoules did not undergo such large temporal variations in quench parameters and *R*_αe_. It may have occurred with only the A and B series samples because of larger quantities of unexpelled HNO_3_ in these more concentrated solutions. The HNO_3_ content of the latter master solution used to prepare the ampoules was diluted by a factor of nearly 150. Nitric acid could very well have a more deleterious effect on the LS cocktails than the HCl diluting solution and could therefore possibly account for the differences observed with the various LS samples. Solution A had a HNO_3_ normality of about 0.35 mol · L^−1^, and solution B may have been comparable if little of HNO_3_ was effectively removed by the gentle heat treatment.

**Fig. 8:**
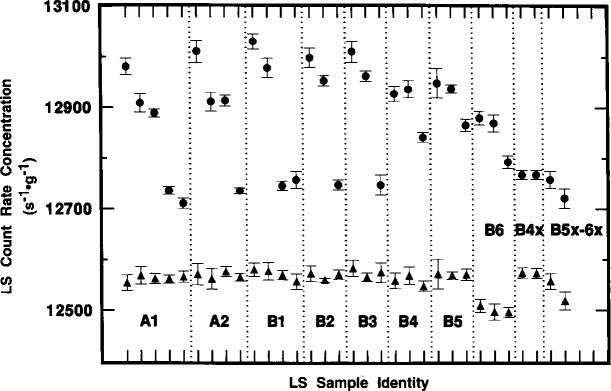
LS counting rate concentrations *R*_αe_ (closed circles) and *R*_α_ (closed triangles) obtained with the Beckman LS system for the A and B series samples. Each plotted value corresponds to the mean for 5 to 10 replicate measurements. The error bars represent standard deviation uncertainty intervals. The multiple determinations shown for a given sample were measurements made at subsequent times (compare Fig. 7 for sample A1).

**Fig. 9:**
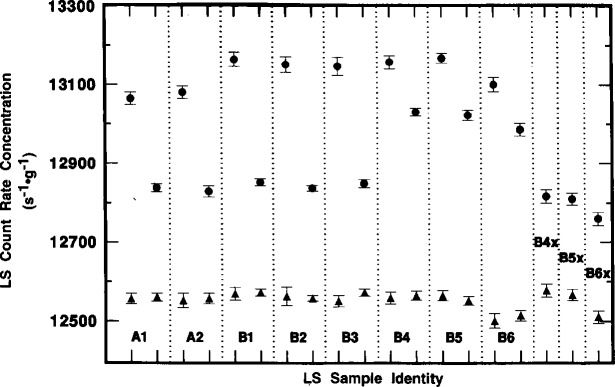
LS counting rate concentrations *R*_αe_ (closed circles) and *R*_α_ (closed triangles) obtained with the Packard LS system for the A and B series samples. Refer to Fig. 8 caption for details.

Although *R*_αe_ was found to vary with time, with sample quenching conditions, and with the instrument used to perform the measurement, the values of *R*_α_ were invariant. Figure 7 illustrates this nicely. The mean values of *R*_α_ for sample A1 over the same time interval are constant and independent of the sample quenching changes. This demonstration is achieved even more powerfully in Figs. 8 and 9 which aggregate all of the measurement results on the series A and B samples. Each plotted value in the figures represents the mean of 5 to 10 measurements of either *R*_αe_ (closed circles) or *R*_α_ (closed triangles). The multiple values shown for a given sample are the mean values for additional 5 to 10 measurement sequences obtained at later times (and with changing quench parameters). The illustrated error bars correspond to the calculated standard deviations for the 5 to 10 replicate measurements. They correspond to *s*_m_ values ranging from 0.02 % to 0.06 %. Figures 8 and 9 give the results obtained with the Beckman and Packard instruments, respectively. Examinations of the figures indicate that although the values of *R*_αe_ vary substantially between instruments, between samples, and at different times for any given sample, all of the *R*_α_ values are essentially constant. This is exactly what is to be expected: invariant and 100 % α detection efficiencies resulting in *R*_α_ values that are independent of quenching conditions or changes; very quench dependent and instrument dependent *R*_e_ values for the low energy ^205^Pb ce; and a greater *R*_e_ response in the instrument with shorter pulse resolving time.

The mean values of *R*_α_ across all samples and measurements are 12560 s^−1^ · g^−1^ for the Beckman (Fig. 8) results, and 12553 s^−1^ · g^−1^ for the Packard (Fig. 9).^3^ The two respective mean values have *s*_m_ values of 0.015 % and 0.017 % based on totals of 197 and 149 replicate measurements. The mean value for all 346 measurements obtained with both instruments is 12557 s^−1^ · g^−1^ with *s*_m_ = 0.016 %. On application of the gravimetrically determined dilution factor *D* = 147.243, the corresponding counting rate concentration for the ^209^Po solution standard is *R*_α_/*D* =85.28 s^−1^ · g^−1^.

#### 2.4.3 M Series Samples

**Fig. 10:**
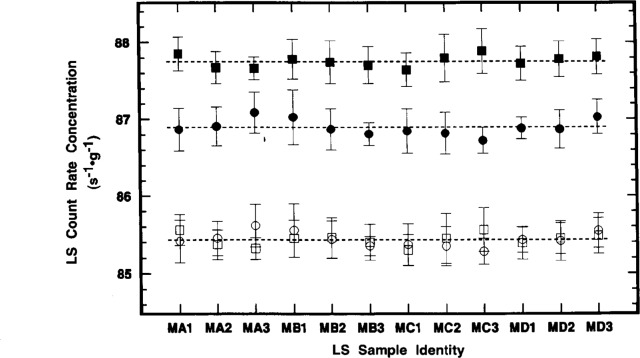
Mean LS counting rate concentrations *R*_αe_ and *R*_α_ obtained with the two LS systems for the M series samples. Closed squares are for *R*_αe_ (Packard); closed circles for *R*_αe_ (Beckman); open squares for *R*_α_ (Packard); and open circles for *R*_α_ (Beckman).

The differences in observed *R*_e_ values obtained with the two LS systems, due to differences in the detection and resolution of the ^205^Pb ce, is even more clearly demonstrated in the results for the 12 M series of samples. The samples (three prepared from each of four randomly selected sealed ampoules) were of closely matched composition and had average quench parameters ranging from *H^#^* = 134 to 135 and *tSIE* = 287 to 291 which did not appreciably alter over a two week period. The observed values for *R*_αe_ and *R*_α_ obtained with the two LS counters are illustrated in Fig. 10. Each plotted determination is based on 10 replicate measurements of each sample on each instrument. The grand means *R*_αe_ (Beckman) = (86.89 ±0.26) s^−1^ · g^−1^ and R_αe_ (Packard) = (87.75 ± 0.24) s^−1^ · g^−1^ differ by about 1 %, while the two grand means *R*_α_ (Beckman) = (85.43 ±0.25) s^−1^ · g^−1^ and *R*_α_ (Packard) = (85.43 ± 0.23) s^−1^ · g^−1^ are in agreement to four significant figures. The above uncertainty intervals given for these means are standard deviations of the means (*s*_m_) with 119 degrees of freedom. These results also demonstrate the excellent between-sample and between-ampoule precision. Table 2 (for the Beckman data) and Table 3 (for the Packard) summarizes the precision estimators *s*_m_ for 10 replicate measurements on any one sample, and for the 3 means of samples prepared from any one ampoule. All of the tabulated mean *R*_α_ values are statistically equivalent, independent of the instrument used, specific sample, or ampoule used to prepare the samples.

#### 2.4.4 N Series Samples

**Fig. 11:**
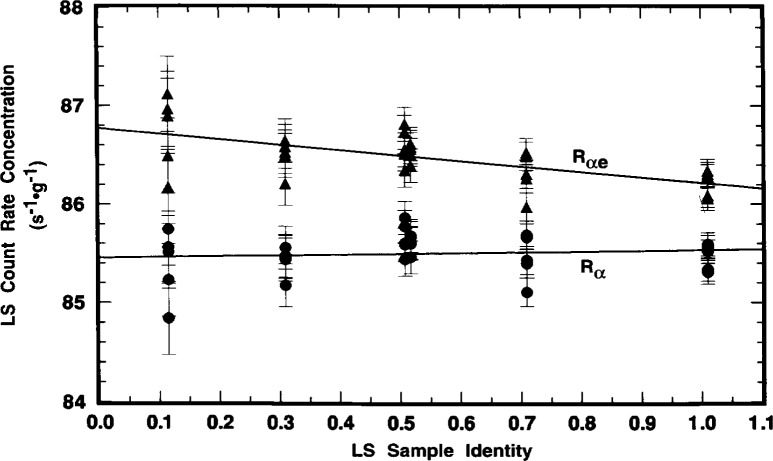
LS counting rate concentrations *R*_αc_ (closed triangles) and *R*_α_ (closed circles) obtained with the Beckman instrument for the N series samples as a function of *m*_s_ (and sample quenching). The solid lines are linear regressions fitted to the data.

**Fig. 12:**
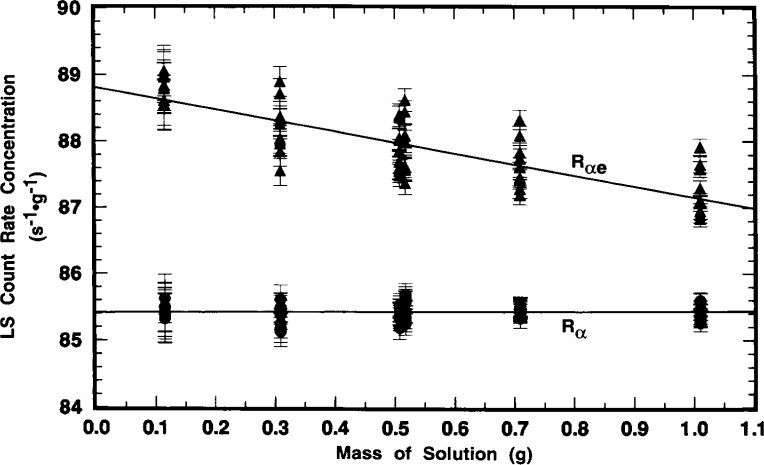
LS counting rate concentrations *R*_αe_ and *R*_α_ as a function of *m*_s_ (analogous to that of Fig. 11) as obtained with the Packard instrument.

The systematic variation of *R*_e_ (obtained from the differences *R*_αe_*−R*_α_) with sample quenching is exhibited in the results of Figs. 11 and 12 for the N series of samples. These data are based on 5 replicate measurements of each sample with the Beckman instrument, and 10 measurements on each sample with the Packard. Their quench parameters were previously provided in Fig. 4. Sample quenching was systematically varied by changing the sample compositions, in this case by varying the mass of ^209^Po solution *m*_s_ used to prepare the samples. Total sample masses and aqueous content *p*_aq_ were kept nearly constant by the additions of blank water (see Table 1). The extrapolations to zero solution mass (*m*_s_ = 0) of Figs. 11 and 12 could equally have been performed using the acid content *N*_aq_ variable. Figures 11 and 12 also illustrate the replicate measurement precision for these LS measurements since all measurement results on each sample are plotted along with their respective error bars that correspond to the total statistical “counting errors.”^4^ The precision is typical of that obtained with all samples prepared from the sealed ampoules.

The weighted *χ*^2^-minimized (least-squares) regression lines shown in Fig. 11 (for the Beckman results) have fitted parameters of:

$$\begin{array}{c} R_{\alpha e}=(86.769\pm 0.083)\;s^{-1}\cdot g^{-1}\\ -[(0.55\pm 0.14)\;s^{-1}\cdot g^{-2}]\;m_{s}, \end{array}$$

and

$$\begin{array}{c} R_{\alpha }=(85.451\pm 0.083)\;s^{-1}\cdot g^{-1}\\ +[(0.077\pm 0.14)\;s^{-1}\cdot g^{-2}]\;m_{s}. \end{array}$$

The linear fits to the data were performed with weighting factors equal to the reciprocal variances 1*/s*_p_^2^(*R*). The fits were performed using all 30 measurement values. The standard deviations on the fitted parameters thus have *v* = (30–2) degrees of freedom. The magnitude and uncertainty on the slope d*R*_α_/d*m*_s_ = (0.08±0.14) s^−1^ · *g*^−1^ clearly indicates that *R*_α_ is invariant of *m*_s_ and of accompanying quench changes. Similarly, the Packard results of Fig. 12 are:

$$\begin{array}{c} R_{\alpha e}=(88.807\pm 0.097)\;s^{-1}\cdot g^{-1}\\ -[(1.65\pm 0.16)\;s^{-1}\cdot g^{-2}]\;m_{s}, \end{array}$$

and

$$\begin{array}{c} R_{\alpha }=(85.415\pm 0.034)\;s^{-1}\cdot g^{-1}\\ +[(0.018\pm 0.057)\;s^{-1}\cdot g^{-2}]\;m_{s} \end{array}$$

with *v* = (60–2) degrees of freedom. The mean *R*_α_ values across all measurements and samples are *R*_α_(Beckman) = (85.492 ± 0.212) s^−1^ · *g*^−1^ and *R*_α_(Packard) = (85.424 ± 0.124) s^−1^ · *g*^−1^. Several distinct observations may be noted. First, the LS response for the ^205^Pb ce (as seen by the *R*_αe_^−^*R*_α_ differences) is greater for the Packard instrument, and is more dependent on changes in sample quenching (as seen by the magnitudes of the fitted slopes d*R*_αe_/d*m*_s_). Second, the magnitudes of d*R*_α_/d*m*_s_ with attendant uncertainties indicate that *R*_α_ is independent of *m*_s_ (and hence quenching). Third, all of the above four *R*_α_ values, whether obtained from the regression intercepts or from the averaging, are statistically equivalent.

### 2.5 1994 4πα LS Measurement Results

The results of 1994 LS measurements, obtained with samples P1 through P5, Q1 through Q4, and Q5 through Q8 (see Table 1) were essentially a redundant and independent reconfirmation of the reanalyzed 1993 results. The LS samples had systematically varied sample quenching using different sample compositions. As indicated in Table 1, some of the sample series used larger scintillator volumes to further minimize quenching. Analysis procedures used to obtain the counting rate concentrations *R*_αe_ and *R*_α_ were identical to those employed with the 1993 data. The LS spectra integrating regions Δ*E*_α_ (in terms of approximate beta energies) were:

**Table:** 

for the Beckman instrument	
Δ*E*_α_≈32 keV to 4400 keV	(P samples)
25 keV to 4400 keV	(Q samples)
and for the Packard instrument	
19 keV to 2000 keV	(P samples)
25 keV to 2000 keV	(Q samples).

Each sample was measured with each instrument from 5 to 18 times. All results were corrected for decay (as well as for the ^208^Po impurity) to the common reference time of 1200 EST 15 March 1994.

#### 2.5.1 P and Q Series Samples

**Fig. 13:**
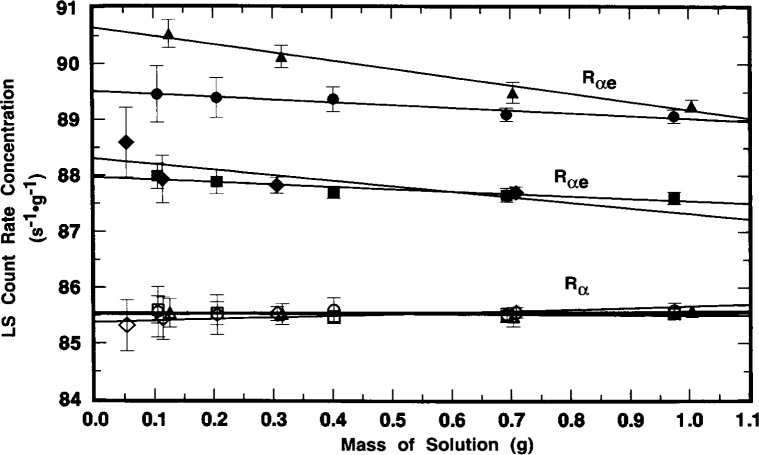
LS counting rate concentrations *R*_αe_ and *R*_α_ obtained with the two LS systems for the P and Q series samples in 1994. Closed squares (*R*_αe_) and open squares (*R*_α_) represent the mean values for samples Q5 through Q8 with the Packard; closed and open triangles represent *R*_αe_ and *R*_α_, respectively, for samples P1 through P5 with the Packard; closed and open triangles (R_αe_ and *R*_α_) are for samples Q1 through Q4 with the Beckman; and closed and open circles (*R*_αe_ and *R*_α_) are for samples P1 through P5 with the Beckman. Each plotted value corresponds to the mean of 5 to 18 replicate measurements on each sample. The error bars represent standard deviation uncertainty intervals on the means. The solid lines are unweighted linear fits to the data. Although the *R*_αe_ values vary with the instrument used to perform the measurements (Packard or Beckman) and with sample compositions, all of the *R*_α_ values are statistically equivalent and invariant.

Figure 13 summarizes the four sets of results obtained with the two LS systems. As previously: (1) *R*_αe_ is invariably larger for the Packard instrument than that obtained with the Beckman (which had a longer pulse resolving time); (2) variations in *R*_αe_ with sample quenching (reflecting differences in detection and resolution of the ^205^Pb ce) is also greater with the Packard; (3) values of *R*_α_ obtained with either instrument are invariant of sample quenching; and (4) the *R*_α_ values for both instruments are statistically equivalent and virtually identical.

Table 4 contains a summary of the various means for *R*_αe_ and *R*_α_, along with their precision estimators, and comparisons of the fitted parameters for the unweighted linear regressions shown in Fig. 13. In these cases, the regressions were performed using the mean counting rate concentrations for each sample. The uncertainties in the fitted parameters thus have *v* = (5 – 2) and *v* = (4 – 2) degrees of freedom for the P and Q sample series, respectively.

### 2.6 LS Spectral Shape Considerations

Extensive examinations of the LS spectra were performed throughout various phases of this work. These examinations involved systematic studies of the detailed shapes and structure of the spectra as functions of the LS sample compositions, chemical quenching changes, and source configurations. They were performed largely in order to understand the observed LS measurement results (particularly in terms of interpretations and compatibility with the ^209^Po decay scheme), and to insure the validity of the analysis procedures used to obtain the previously given *R*_α_ values.

#### 2.6.1 Quench-Dependent Gross Effects

**Fig. 14:**
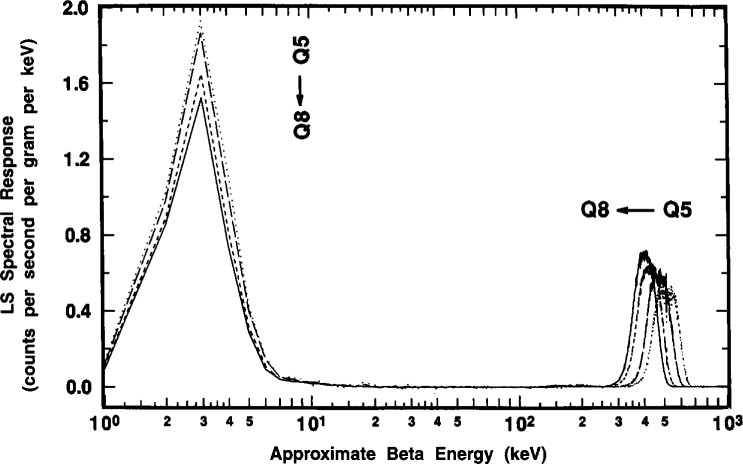
LS spectra of increasingly quenched samples Q5 through Q8 obtained with the Packard counting system.

Typical gross effects of chemical quenching on the ^209^Po spectrum are illustrated in Fig. 14 for the Q5 through Q8 samples. The four spectra were obtained with the Packard instrument. The quench parameters varied from *tSIE* = 528 for least-quenched sample Q5 to *tSIE* =414 for Q8 and were determined with a precision having a relative standard deviation of the mean of 0.16 % to 0.17 %. Two dominant features can be noted. With increasing quenching in the samples (i.e., decreasing *tSIE* values), the broad α peaks at around an equivalent beta energy of 350 keV to 550 keV shift to lower energies, and the integrated areas of the low-energy ce peaks progressively decrease in magnitude. These latter peak areas are, of course, merely the *R*_e_ values as given, for example, by the changing (*R*_αe_*−R*_α_) differences in Fig. 13. The α peak areas (given by *R*_α_ in Fig. 13), although shifted to different energies, are invariant of the quenching changes.

**Fig. 15:**
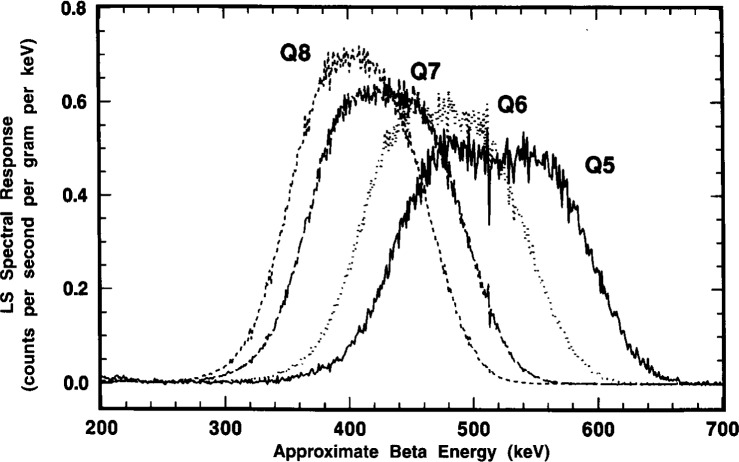
Details of the broad alpha peaks shown in the full spectra of Fig. 14. The peak widths (FWHM) on a relative basis and peak areas are approximately equal in all four samples Q5 through Q8.

These α peak shapes are considered in greater detail in Fig. 15. One may observe that the spectrum for the least quenched Q5 sample almost has the appearance of an unresolved doublet. This feature was also seen (at times in this laboratory, as well as by other researchers) in other LS spectra from monoenergetic α-emitting radionuclides, e.g., ^208^Po [3], and is frequently attributed to “optical effects” arising from refractions of the light photons in the LS vial walls, or from light reflections in the LS counting chamber or off the vial walls. Similar “doublets” were also observed however, in the earlier 1993 spectra obtained with polyethylene vials, and was seen in spectra from both the Beck-man and Packard LS counting systems. These were surprising findings. One would think that the glass and polyethylene vials have considerably different refraction and surface reflection properties; and that there undoubtedly are some differences in the “optics” for the two counting systems. This “doublet” effect is rarely apparent in more highly quenched samples when the alpha peak shifts to lower energies as shown in Fig. 15.^5^ With the shifts to lower energies, the resolution (or peak widths) also decrease on an absolute basis. The peak widths, given by full-width-at-half-maximum (FWHM) values, for the four spectra of Fig. 15 range from about 120 keV to 170 keV. On a relative basis compared to the peak centroids, however, all four have FWHM values of approximately 30 % (within 1 % or 2 %).

#### 2.6.2 “Anomalous Bumps”

Another surprising feature of the LS spectra is the presence of “anomalous bumps” distinctly separated from the a peaks and at energies just below these peaks. These bumps were actually identified prior to understanding the detection of the delayed 2.3 keV ce when the LS results still seemed inexplicably discrepant. It was thought that perhaps the differences in the initially observed *R*_αe_ counting rates between the two LS counters might be due to these mysterious bumps that could not be attributed to any known transitions in ^209^Po decay. As a result, the spectral structures and magnitudes of these bumps were extensively examined and systematically studied. They were observed in all of the LS spectra, in all of the samples ranging from the 1993 A, B, M, and N series of samples to the 1994 P and Q sample series (which therefore encompassed use of both polyethylene and glass LS vials), and in both counting systems.

**Fig. 16:**
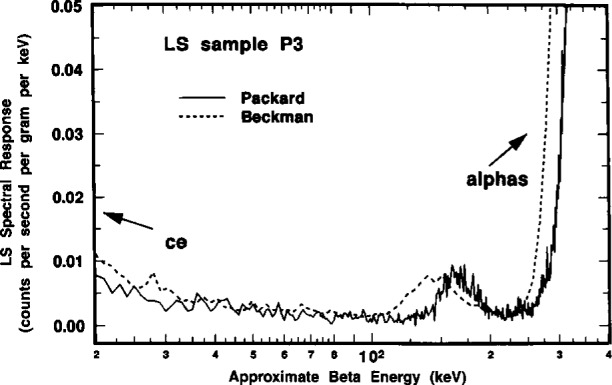
Comparison of the “anomalous bumps” in the spectra for sample P3 obtained with the two LS counting systems.

**Fig. 17:**
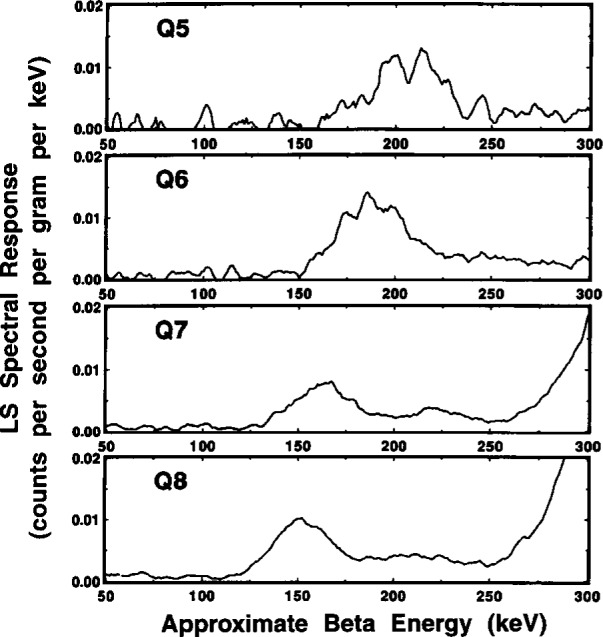
Comparison of the “anomalous bumps” in the LS spectra of four samples having different quench conditions.

Figure 16 contains a typical example of these “anomalous bumps” as seen in spectra of sample P3 obtained with both spectrometers. The bumps, like alpha peaks, shift to lower energies with increased quenching as seen in the series of spectra in Fig. 17. In this case, the bump centroids decrease from about 210 keV in the least quenched sample Q5 to about 150 keV in sample Q8. The data of Fig. 17 (unlike all other displayed LS spectra) were smoothed using an averaging Savitsky-Golay differentiation technique to eliminate the wide fluctuations in the data (arising from large variations in the small counting rates per channel and their inherently large statistical “counting errors”) and thereby to more clearly show the bump structures. The shifts of the bumps to lower energies were not found to be systematically correlated to the α peak shifts. About the only conclusive statement that can be made is that the energy differences in the bump shifts were less than that for the shifts in the α peak centroids for given quench changes. If a correlation exists, it is not a simple relationship. There were also suggestions that the bumps became more dispersed and broadened (on both an absolute and relative basis) with increasing sample quenching, and that perhaps even secondary bumps appeared in the valleys between the principal bump and the α peak (see, for example, the spectra for Q7 and Q8 in Fig. 17). The relative magnitudes of the bumps in comparison with the α peak (in the same spectrum) were highly variable, ranging from less than 0.32 % to over 2.2 %. Again, there was no obvious correlation or simple relationship between the magnitude of the bumps and any sample quenching or sample counting conditions.

For reasons outlined immediately below, it is believed that these anomalous bumps are most likely due to optical effects from α interactions in the cocktails, and not due to LS responses from some other non-α transitions. Why, how, when, and where they appear in the spectra, however, still remains unknown. Sample preparation effects due to polonium in a two-phase condition can not be ruled out as the causal factor. This would be surprising however considering the presence of the bumps over such a wide range of sample compositions and conditions. It should also be emphasized that if these bumps were not included as part of the 4πα LS response, then the *R*_α_ values given previously would not have formed a quench-independent and consistent data set.

#### 2.6.3 Alpha-Beta Pulse Discrimination

Several LS spectra obtained using alpha-beta (α-β) pulse discrimination were also measured to insure that the previously ascribed spectral interpretations were valid, particularly in regard to the assignments of the “anomalous bumps” to α interactions and of the very low ≈3 keV peak to conversion electrons.

The LS counting sources used to obtain these spectra consisted of about a 1 g aliquant of the ^209^Po standard solution (obtained from a randomly selected sealed ampoule, #146) in about 14 g of a prepared scintillation fluid. The scintillating cocktail was “Ultima Gold AB” (Packard Instrument Co.) which was selected because it provides slow pulse decay characteristics which are necessary for effective α-β pulse discriminations. The prepared scintillator is also reported to be very efficacious for accepting high mineral acid loadings (up to 2 mL of 2 mol · L^−1^ HNO_3_ in 10 mL of scintillator). It may be noted that the previously used “Ready Safe” scintillation cocktail did not provide adequate α-β pulse discrimination.

The spectra were obtained with a slightly different model of the Packard Tri-Carb 2500TR LS spectrometer that was used for all of the previously described Packard instrument measurements. Pulse discrimination between α and β pulses relies upon their differing pulse decay times. Initial spectra were taken with a nearly arbitrarily chosen mid-range pulse discrimination setting with the expectation that there would be some accidental overflows of α pulses in the β spectrum, and vice versa.

**Fig. 18:**
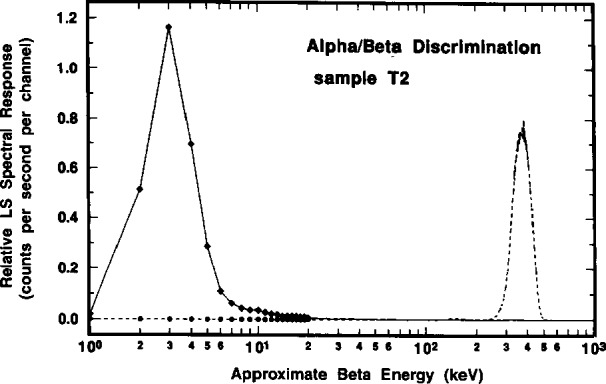
LS spectrum of ^209^Po obtained using alpha and beta pulse discrimination.

**Fig. 19:**
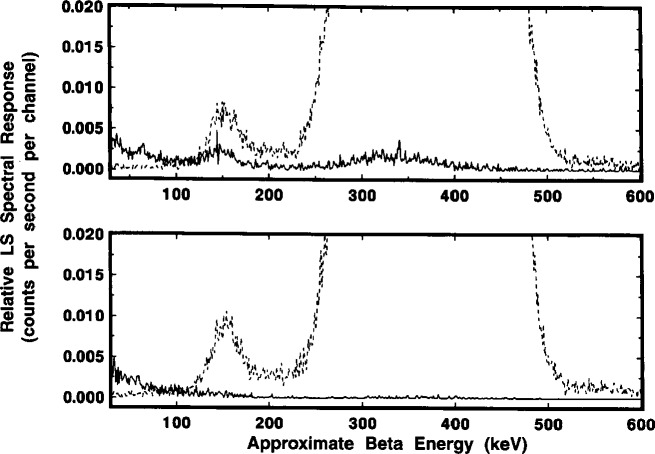
Details of the LS spectrum of Fig. 18 showing the relative alpha and beta responses for the “anomalous bump” and α peak at two pulse discrimination settings.

Figure 18 gives a composite of the respective α spectrum (broken line) and β spectrum (solid line) obtained with the initially chosen discriminator setting. As shown, the low energy peak centered at around 3 keV is clearly due to β (i.e., electron) pulses; whereas α selected (and β discriminated) pulses clearly comprise the ascribed broad α peak. Expanded scale details of these spectra in the energy interval of 50 keV to 600 keV are given in the upper trace of Fig. 19. Of the intense α peak, only a very small fraction of the response is from β selected pulses. These are attributed to “accidentals” whose relative magnitude was dependent on the specific discrimination setting used. At this same setting, less than 20 % of the total area of the “anomalous bump” was due to selected β pulses. Spectra obtained with a 25 % change in the initial α-β pulse decay discrimination setting resulted in those in the lower trace of Fig. 19. In this latter case, the accidentals under the α peak dramatically decreased and virtually the entire anomalous bump is comprised of selected α pulses. The findings reinforced the belief that the anomalous bumps were indeed the result of some unknown optical effects in detecting some small fraction of the α interactions.

#### 2.6.4 Optical Effects in Differing Counting Source Configurations

Lastly, in an attempt to discern (if not understand) the possible optical effects, several spectra were obtained using sources of very different counting configuration. It was thought that the “optics” of these configurations would of necessity be substantially different. The configurations consisted of a normally prepared sample source containing *m*_tot_≃11 g of scintillator and sample in a 22 mL glass LS vial (nominal 2.5 cm diam.); a source containing *m*_tot_≃23 g of scintillator and sample in a similar 22 mL glass vial such that the vial was completely filled with cocktail; and another completely filled source in a smaller 1.4 cm diameter glass vial that was in turn concentrically contained in a C_6_H_14_ (hexane) filled 22 mL vial. The sources are designated N (for “normal”), F (for “full”), and D (for “double”), respectively. The sources were prepared with closely matched chemical quenching conditions, and the spectra obtained with them were normalized to the ^209^Po solution mass contained in each. In this way, if the sources had been in identical configurations and with identical optics, then one could expect identical spectra for them.

Comparison of the N and D sources was made to see if the “anomalous bumps” were at all associated with the presence of an air space in the N source configuration. Conceptually, it was thought that perhaps a lower energy α peak might arise because of scintillations from any cocktail (above the air gap) that adheres to the vial cap surfaces. The filled configuration of source D would obviate this condition, although there would of necessity be some loss of light from scintillations occurring in the cocktail volume above the vial cap. Comparisons were made with the D source configuration since it is known that the resolution of α spectra can be improved by decreasing the sample source cross-sectional area (e.g., by using smaller diameter sample tubes) [22]. The smaller diameter vial of source D was placed inside the larger 22 mL vial to keep the total source volume constant. The glass wall of the inner vial was virtually transparent since it was surrounded by hexane, which has a nearly identical refraction index as the scintillation cocktail.

**Fig. 20:**
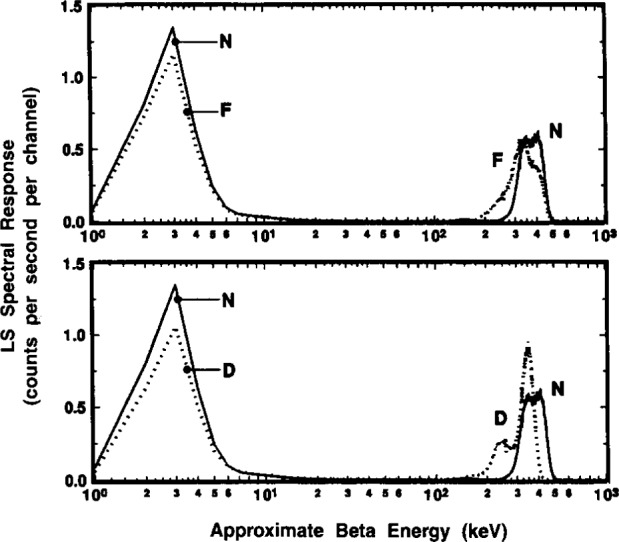
Comparative ^209^Po LS spectra for three very different source configurations N, F, and D. Refer to text for details. The upper trace compares the spectra for N and F; the lower compares N and D.

**Fig. 21:**
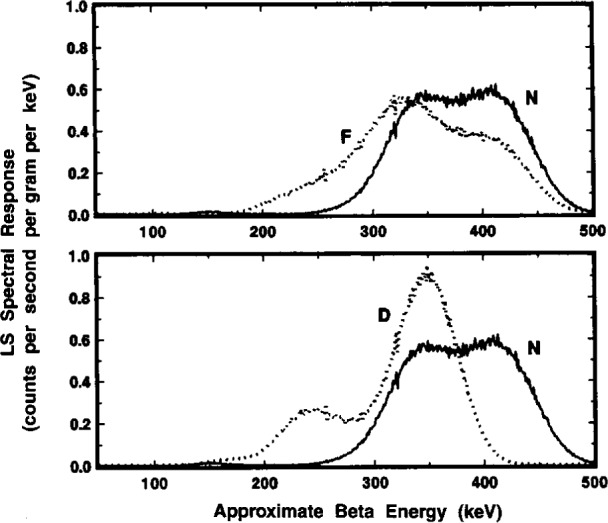
Details of the broad alpha peaks for the three source configurations shown in the spectra of Fig. 20.

**Fig. 22:**
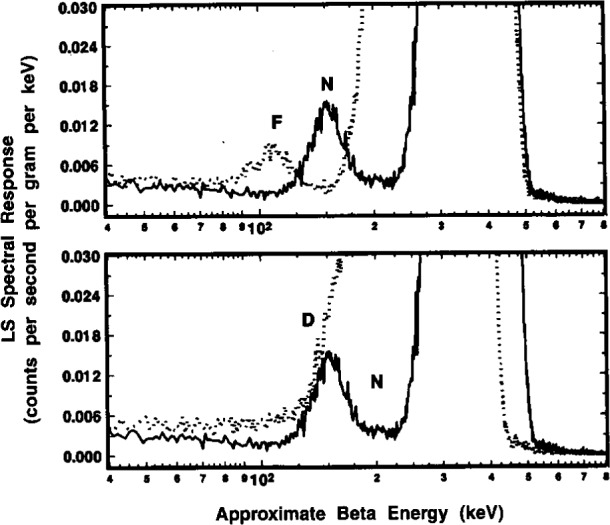
Details of the “anomalous bumps” for the three source configurations shown in the spectra of Fig. 20.

The results of these comparisons are shown in the spectra of Figs. 20, 21, and 22. The first (Fig. 20) provides the full spectra including the low energy ce peaks. The second (Fig. 21) gives details of the α peaks. The broad α peak tailing into the “anomalous bump” region is shown lastly (Fig. 22). The integral intensity of the ce peaks progressively decreases in going from the N to F to D configurations. This may partly be due to light losses above the vial caps in the fully filled F and D configurations. The N configuration shows the apparent “doublet” nature of the broad α peak and the by-now consistent “anomalous bump.” In source configuration F, the bump is still present but is of less intensity and is shifted to a lower energy. At the same time, the α peak from source F has substantially broadened in comparison to source N, and has even more of the appearance of an unresolved doublet with a very long low energy tail. The most surprising results were that for source configuration D. The α resolution with a peak centered at 350 keV was as expected considerably improved with a FWHM of about 70 keV which is less than half that obtained with configuration N. Yet, this α peak is also accompanied by another large and adjoint secondary α peak centered at 250 keV. This secondary peak encompasses a large fraction of the total α response (about 1/4) and also contained very long low energy tailing. The spectra of configuration D did not exhibit any of the usually-found “anomalous bumps,” but the tailing on the secondary α peak had a discrete inflection point. All of the above described spectral structures (in every source configuration) appear, in fact, to be nothing but a complex variety of shape changes (of a continuous nature) in the α peak.

These spectral shape findings can not be explained (at least not by the present authors) in terms of any overall qualitative theory or model for the scintillation optics. The findings, however, tended to support previous contentions that the “anomalous bumps” were associated with true α interactions in the scintillator, and that they arise from some type of light-collection optical effects that in turn could be influenced by changes in source configuration.

### 2.7 Confirmatory Measurements by 2πα Proportional Counting

Independent confirmatory measurements of the ^209^Po α emission rate concentration were performed by 2πα proportional counting of solid point sources LX1 and LX2. The detector consisted of a 12 cm diameter hemispherical gas-flow proportional counter. The counting gas was a “P-10” argon-methane (90:10) mixture. A detailed description of the detector and its operation has been previously described by Hutchinson [23].

**Fig. 23:**
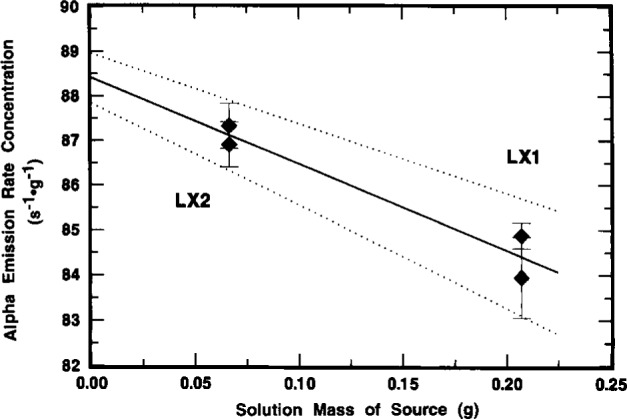
Confirmatory measurements of the ^209^Po α-emission concentration by 2πα gas-flow proportional counting. An extrapolation to zero source mass (without backscattering corrections) is given by a linear fit to the data (solid line). The broken lines are the uncertainty limits for the regression.

The results of duplicate measurements of each source are given in Fig. 23. The illustrated α emission rate concentrations as a function of source mass were obtained from the observed net counting rates (after background subtraction) which included an extrapolation to zero pulse height [23]. The corrected net rates were converted to concentrations by applying the known solution masses that were used to prepare the sources. The detection efficiency was assumed to be exactly 0.5 counts per alpha for a perfect 2π steradian solid angle, exclusive of source self-absorption losses and source-mount back-scattering effects. The relative standard uncertainty in the 2π efficiency is typically taken to be ±2.5 % [23]. The sources had been measured in mid-June 1993, but the values reported here were decay corrected to the 15 March 1994 reference time for direct comparison with the LS measurement results. These values also contain a small correction for the known ^208^Po impurity (including its decay) so that they correspond to only the ^209^Po α emission rate concentration. The error bars shown on the data of Fig. 23 represent one standard deviation uncertainty intervals for the assumed Poisson-distributed total statistical “counting error.” These uncertainties ranged from ±0.3 % to ± 1.1 % for the four determinations.

Figure 23 also contains an extrapolation of the α emission rate concentration to zero source mass to account for source self absorption. The solid line corresponds to an unweighted *χ*^2^-minimized linear regression fitted to the data. The uncertainty bounds for the fit are shown as dotted lines. The fitted intercept (at zero source mass) for the α emission rate concentration is (88.41 ±0.56 s^−1^ · g^−1^.^6^ Using a weighted linear regression (with weighting by the reciprocal of the Poisson “counting error” variance), the fitted intercept **is** (88.52 ±0.52) s^−1^ · g^−1^ which is indistinct from that for the unweighted fit. For “weightless” sources mounted on platinum, the backscattered fraction in 2π has been shown to be 0.0175 ±0.0025 [24,25]. Invocation of this 3.5 % back-scattering correction results in a corrected value of 85.5 s^−1^ · g^−1^ for the ^209^Po α emission rate concentration for the solution standard. These applied corrections for self absorption (by extrapolation to zero mass) and back scattering (which assumed a condition of source weightlessness) are of course somewhat simplistic. In actuality, the source mass extrapolation of Fig. 23 included some part of backscattering effects since the magnitude of any source mount scattering will of necessity by dependent on the source mass itself. The two effects are complexly related. Nevertheless, the relative standard uncertainty in the α emission rate concentration may be considered to be roughly about ± 3 %, considering the uncertainties in the fitted intercept and in the 2πα detection efficiency (including the source absorption and scattering effects). Clearly, the 2πα measurements, although less accurate, admirably served as a suitable confirmatory determination that was completely independent of the LS measurement results.

### 2.8 Alpha-Emission Impurity Analyses

Analyses for α-emitting radionuclidic impurities were also made with the LX1 and LX2 point sources. The analyses were performed by α spectrometry using a Si surface-barrier junction detector which was suitably coupled to a 1024-channel pulse-height analyzer. Conversion gain settings were typically 5 keV to 10 keV α energy per channel. The sources were measured through a very thin film absorber to prevent surface contamination of the detector face. The film consisted of VYNS, a polyvinylchloride—polyvinylacetate copolymer, of nominal surface density ≂10 μg · cm^−2^ that was stretched on an aluminum annular support ring. Multiple spectra with counting time intervals ranging from 2 × 10^4^ s to 6 × 10^5^ s were obtained over the course of 26 d in the time period of May to June 1993.

**Fig. 24:**
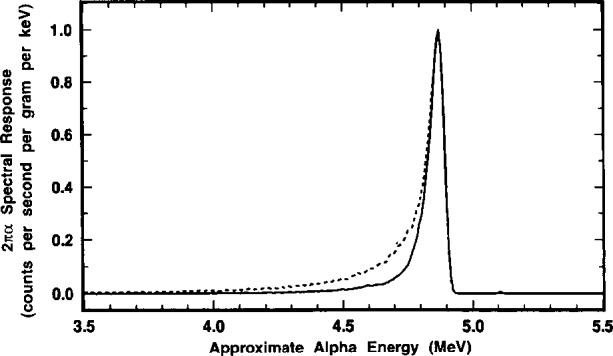
Comparative α spectra for sources LX1 and LX2 obtained with a Si surface-barrier junction detector. The spectra were normalized to the same peak heights to show the greater low-energy tailing in the more massive LX1 source.

Typical spectra for the two sources, showing the partial region for the principal ^209^Po 4.883 MeV α transition, are given in Fig. 24. The effective energy resolution for the detector/source combination, in terms of the peak full width at half maximum (FWHM), is roughly 50 keV to 60 keV. This can be contrasted to an optimum 10 keV to 30 keV which would be expected from a “weightless” source without a backing mount. Low energy tailing, resulting from degradation of the α full energy as well as from some possible small influence of the unresolved ^209^Po 4.622 MeV a, is evident in the spectra from both sources. The weak 4.622 MeV α [*I*_α_ = (0.0092±0.0005) alphas per decay] is completely masked by the more dominant 4.883 MeV peak. The low-energy peak broadening and observed FWHM energy resolution can not be attributed solely to ^209^Po source material self absorption since the solution deposits used to prepare the sources were carrier free and therefore should have been nearly “weightless” after drying. More probable contributing factors were: inhomogeneous source depositing; deposits of unknown chemical impurities (most likely Pb and Bi); backscattering from the thick Pt source mounts; surface irregularities in the back mounts; and absorption in the VYNS barrier.

**Fig. 25:**
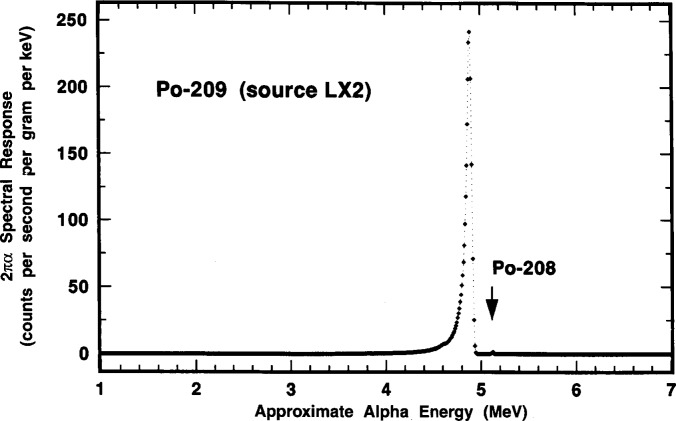
Detail of the ^209^Po α spectrum showing the relative magnitude of the ^208^Po impurity. The spectrum was obtained with source LX2 and the Si surface-barrier junction detector.

**Fig. 26:**
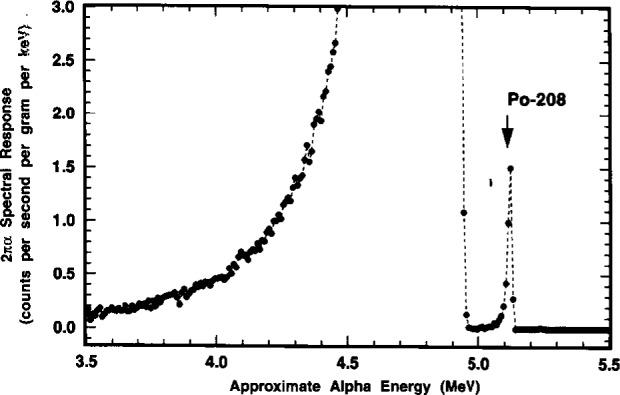
Expanded scale detail of 5.115 MeV α from the ^208^Po impurity in the ^209^Po α spectrum of Fig. 25.

The only observed α-emitting impurity, based on a total cumulative counting time exceeding 1.6×10^6^ s (≈19 d), was ^208^Po whose principal α (*I*_α_ = 0.999982±0.000002) has an energy of *E*_α_ = (5.1152±0.0015) MeV [11]. There was no evidence of ^210^Po presence *[E*_α_ = (5.30438±0.00007) MeV; *I*_α_ = 1.00] [26,27] which is unsurprising because of its substantially shorter half-life *(T* = 138.4 d) and age of the ^209^Po stock material. Detection of these two most likely polonium isotope impurities is greatly facilitated in that their principal α transitions have energies greater than that for ^209^Po. Figures 25 and 26 illustrate the relative ^208^Po and ^209^Po α intensities. The former, in full scale, clearly shows the large relative magnitude difference in the two peaks, although that for ^208^Po is barely perceptible; the latter, in an expanded scale, demonstrates the distinct resolution and definition of the 5.115 MeV α from the ^208^Po impurity.

Three independent determinations of the ^208^Po to ^209^Po impurity ratio, in terms of their respective α-particle emission rate concentrations, were performed. The results, which were decay corrected to the common reference time of 1200 EST 15 March 1994, are summarized in Table 5. The propagated relative uncertainties for the “counting errors” and for location of peak backgrounds ranged from ±1.6 % to ±3.9 % and from ±1.6 % to ±2.1 %, respectively. The ^208^Po/^209^Po impurity ratio mean for the three determinations was 0.00124 with a standard deviation of 0.00010 (or ±8.1 % on a relative basis).

Further examinations of the spectral backgrounds in certain energy regions could establish limits for the presence of any other α emissions. Impurity ratios, in terms of emission rate concentrations X_α_*/*^209^Po, for α particles of energy *E*_α_ (arising from an α-emitting impurity X_α_ in the ^209^Po standard solution) have the following lower limits: in the region *E*_α_>5.18 MeV (which includes ^210^Po), X_α_/^209^Po≤2×10^−6^; in the region 3.5 MeV≥*E*_α_≤ 4.2 MeV, X_α_/^209^Po≤6×10^−4^; and in the region *E*_α_≤3.5 MeV, X_α_/^209^Po≤2×10^−5^.

### 2.9 Photon-Emission Impurity Analyses

Analyses for photon-emitting radionuclidic impurities were performed with samples Gl through G3 using two different spectrometry systems. The systems comprised (a) an intrinsic p-type Ge detector (having a NaI(T1) equivalent efficiency of 18 %) coupled to a 16384-channel pulse height analyzer (PHA) that spanned a 40 keV to 3300 keV energy region; and (b) an intrinsic n-type Ge detector (12 % NaI(T1) equivalent efficiency) which used a 8192-channel PHA spanning 8 keV to 915 keV. Both systems employed a 60 Hz puiser for rate normalizations. Detection efficiency curves for the systems were determined with calibrated (NIST SRM) ^241^Am, ^111^In, ^57^Co, ^51^Cr, ^85^Sr, ^137^Cs, and ^60^Co sources.

Photons associated with both the EC and α branch decays of ^209^Po include Bi K x rays arising from K capture in the EC branch and from internal conversion fluorescence of the 896.6 keV transition in ^209^Bi; the 896.6 keV γ ray in ^209^Bi; Pb K x rays from internal conversion of the 260.5 keV and 262.8 keV γ rays in ^205^Pb; and the 260.5 keV and 262.8 keV γ rays in ^205^Pb (see Fig. 2). All of these photons, including partial resolutions of the Pb and Bi K_α1α2_ and K_β1β2_ x rays, were detected. Determinations of their absolute intensities, based on the present ^209^Po calibration, will be reported separately [28] in a re-evaluation of the ^209^Po decay scheme.

No photons other than those associated with the decay of ^209^Po were detected. Emission rate concentrations^7^ for any other photons arising from photon-emitting impurities in the ^209^Po standard solution have the following lower limits: <2×10^−4^ s^−1^ · g^−1^ in the energy region 15 keV to 68 keV; <1.5×10^−4^ s^−1^ · g^−1^ in the region 81 keV to 256 keV; <6×10^−5^ s^−1^ · g^−1^ in the region 266 keV to 892 keV to; and <4×10^−6^ s^−1^ · g^−1^ in the region 900 keV to 3300 keV, provided that the energy of any such unobserved photon is 4 keV or more different from the emissions accompanying the ^209^Po decay. These limits were based on numerous independent measurements of the sources with the two spectrometer systems over a total counting time interval of 7.2 × 10^6^ s (≃83 d).

### 2.10 Calibration Summary and Uncertainty Analysis

#### 2.10.1 Mean LS Counting Rate Concentration

The LS determinations of *R*_α_ obtained with both counting systems and with all of the 1993 and 1994 samples are summarized in Table 6. The means given in Table 6 were obtained from unweighted averages across all measurements in the given sample series. Weighted means were generally indistinguishable since reciprocal variance weighting factors were all of the same nominal magnitude.

As shown previously in Figs. 8 through 13 and Tables 2 through 4, the mean value of *R*_α_ for any sample within a given sample series and obtained with a given instrument was statistically equivalent (with the possible exception of sample B6—see Figs. 8 and 9) with that for any other sample. Similarly, as shown in Table 6, when these sample means are averaged over a given sample series, the resulting means for any two sample series were also equivalent. Further, the means obtained with either the Beckman instrument or the Packard were invariant.

The ratio of mean *R*_α_ for the A and B sample series to the mean *R*_α_ for the M through Q sample series may be directly compared to the gravimetric dilution factor. These are:

$$\begin{array}{c} D\;(\text{Beckman})=\frac{(12560\pm 27)\;s^{-1}\cdot g^{-1}}{(85.469\pm 0.244)\;s^{-1}\cdot g^{-1}}=146.95\pm 0.52\\ D\;(\text{Packard})=\frac{(12553\pm 26)\;s^{-1}\cdot g^{-1}}{(85.466\pm 0.209)\;s^{-1}\cdot g^{-1}}=146.88\pm 0.47 \end{array}$$

compared to *D* (gravimetric) = 147.24 ± 0.07 which is approximately 0.2 % larger. Although they are in statistical agreement, the absolute differences are slightly larger than that usually obtained by our laboratory for verifications of gravimetric dilution factors. Typically, LS verification measurements of dilution factors are $\tilde{<}0.1\%$. As noted previously, the differences may in part be due to chemical quenching differences arising from greater quantities of HNO_3_ in the A and B sample series. Nevertheless, these observed differences are irrelevant insomuch as the ^209^Po calibration can be based directly on the measurements of samples (sealed ampoules) of the master solution.

The overall results of Table 6 may be averaged in several ways. Consideration of the possible mean values for *R*_α_ derived in these different ways is informative. With the Beckman instrument, the mean *R*_α_ for all 27 samples of the M through Q sample series, involving a total of 260 independent LS determinations of *R*_α_ is (85.469 ± 0.244) s^−1^ · g^−1^. The above standard deviation has a corresponding relative standard deviation of the mean of *s*_m_ = ±0.018 %. For the Packard instrument, based on 276 measurements with 27 samples, the mean *R*_α_ is (85.466 ±0.209) s^−1^ · g^−1^ (*s*_m_ = 0.015 %). As noted, these aggregated means can be compared to that obtained from replicate measurements on any one sample (Tables 2 and 3; and Figs. 8 through 13), as well as to the means obtained from all samples within the various sample series (Tables 2 through 4, and 6). They are all equivalent. One could alternatively average across the means obtained from the results from each of the seven ampoules to obtain grand means. These are (85.460 ± 0.063) s^−1^ · g^−1^ (*s*_m_ = 0.024 %) and (85.460 ± 0.055) s^−1^ · g^−1^ (*s*_m_ = 0.021 %) for the Beckman and Packard results, respectively. The precision estimators on these grand means reflect the between-ampoule variability. Lastly, the overall mean obtained from all 536 determinations (from both LS instruments) on all 27 samples from the 7 different ampoules is *R*_α_ = (85.468±0.227) s^−1^ · g^−1^ (*s*_m_ = 0.012 %).

It is significant to realize that the 536 measurement results embodied in this last mean were obtained under a tremendous range of conditions. They were obtained: (i) with two different counting systems; (ii) using different Δ*E*_α_ integrating regions; (iii) with different counting time intervals; (iv) from measurements separated by more than a year in time; (v) with background subtraction variabilities over this time (vi) with decay corrections for ^209^Po as well as for the ^208^Po impurity over this same time; (vii) using counting sources in both glass and polyethylene LS vials; (viii) with sources prepared from 7 randomly selected ampoules of the ^209^Po standard solution; and (ix) with 27 samples having wide variations in sample composition (including aliquant size of the ^209^Po solution and hence emission rates) and chemical quenching conditions.

The results of these determinations of *R*_α_ were also subjected to extensive statistical tests on subsets of the data across a variety of variables: sample identity including sample preparation order, sample composition, sample masses, sample replicates for a given ampoule of the ^209^Po solution, the timing and sequence of the LS measurements, and the LS measurement system used. These included *χ*^2^-tests of the homogeneity in subsets of the observed sample variances (across the variables), *F*-tests of the homogeneity in the various subset sample means, *t*-tests of differences between the various subset means, and tests of possible correlations and biases using analysis of variance (ANOVA) techniques with sequential two-way classifications of the variables. None of the tests indicated any statistically significant differences in any of the tested subset sample means or sample variances. The agreement in the results between the various sample series and between the two LS counting systems, as provided in Table 6, are representative.

**Fig. 27:**
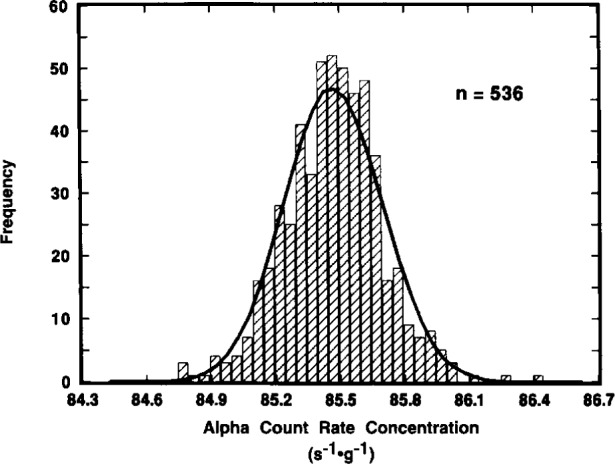
Frequency distribution for the 536 determinations of the LS counting rate concentration *R*_α_. The solid line is for a normal distribution having parameters of *μ* = 85.462 s^−1^ · g^−1^ and σ = 0.227 s^−1^ · g^−1^.

The 536 values comprised in the mean *R*_α_ = (85.468±0.227) s^−1^ · g^−1^ are normally distributed. This is demonstrated in Fig. 27 where a frequency distribution of the data is superimposed on a Gaussian (normal) probability distribution function (solid line) having parameters of *μ* = 85.468 and σ = 0.227.^8^ The probability function was normalized to the *n* = 536 sample size so that both distributions have equal areas.

#### 2.10.2 α Emission Rate Concentration

The ^209^Po α-particle emission rate concentration was obtained from the mean *R*_α_ on application of the two aforementioned correction factors, viz., *A*_α_ *= R*_A_*k_Z_*_E_*k*_EC_.

The first, *k*_ZE_, was found to be highly variable from spectrum to spectrum because of the low counting statistics and imprecision in locating a baseline under the ce peak in extrapolating to zero energy. As a result of this variability, it was decided to apply an average correction to all of the spectra. Values of *k*_ZE_ ranged from lows of *k*_ZE_≃1.0002 to highs of *k*_ZE_≃1.0008, with a majority centering around a value of *k*_ZE_≃1.0006 (i.e., the integral area for the extrapolated baseline under the ce peak was 0.06 % of *R*_α_). No obvious correlation between the observed magnitudes of *k*_ZE_ and quenching could be found. The estimated standard uncertainty on the central value of 1.0006 was taken to be ±0.0003 (i.e., ±0.03 % on a relative basis). There is an additional uncertainty, estimated to be ±0.05 %, for extrapolation to zero pulse height. The relative combined standard uncertainty in *k*_ZE_ is thus taken to be ± 0.06 %, such that *k*_ZE_ = 1.0006±0.0006.

The second correction factor, *k*_EC_ = 0.9988 ± 0.0006, is for the contribution to the LS response from radiations that arise from the ^209^Po EC decay. The above value was obtained from approximations given in Appendix A.

The mean α emission rate concentration is therefore *A*_α_ = 85.42 s^−1^ · g^−1^ (as of the 1200 EST 15 March 1994 reference time). Assuming an α-decay branching ratio of *I*_α_ = 0.9952 ± 0.0004 from Martin [11], the corresponding ^209^Po activity concentration is 85.83 Bq · g^−1^.

#### 2.10.3 Uncertainty Assessment

The relative combined standard uncertainty in *A*_α_ is *u*_c_ = ±0.17 %. The relative expanded uncertainty, using a coverage factor of *k* = 2, is *U* = ± 0.34 % which is assumed to provide an uncertainty interval of 90 % to 95 % confidence. The component uncertainties used to obtain these estimates are outlined in Table 7. These component uncertainties are given in terms of their contribution to the *relative* standard uncertainty in *A*_α_, i.e., $u_{A_{\alpha }(j)}=[(\partial_{A_{\alpha }}/\partial_{z_{j}})u_{z_{j}}z_{j}]/A_{\alpha }$ for each component *z_j_* having a *relative* uncertainty estimate of $u_{z_{j}}$. Of necessity, some of the uncertainty components are difficult to quantify on a purely objective basis and are therefore based on subjective experiential estimates. Analyses, justifications, and underlying assumptions used to quantify all of the components follow.

Component (a), the first in Table 7, is the LS measurement imprecision given by the relative standard deviation of the mean *s*_m_ = 0.012 % for the 536 independent determinations of *R*_α_. It, of necessity, contains random variabilities (wholly or in part) from many component sources of inaccuracy: background subtraction variabilities, spectral integrations, LS cocktail stability, chemical quenching, timing errors, sample mass determinations, and decay corrections. For comparison, the *s*_m_ for 10 replicate measurements on a single given sample typically ranged from 0.05 % to 0.1 %; and *s*_m_ for all replicate measurements within a given sample series was typically ± 0.05 % (see Tables 2 through 4).

Component (b) is the uncertainty in mean *A*_α_ for any given ampoule of the ^209^Po solution standard. It was obtained from the standard deviation for the between-ampoule dispersion (see Sec. 2.10.1) and has ν = (7 ampoules−1) = 6 degrees of freedom. It also of necessity contains inherent random variabilities from other uncertainty components.

Component (c) is an estimate of the uncertainty in the LS detection efficiency, and its variability with chemical quenching and over the observed system variations (for all of the variables of these measurements). The estimate was approximated in several ways. The first, call it (c1), was obtained by comparing the mean *R*_α_(mean) for all samples and measurements within a given sample series (and with a given LS spectrometer) to the extrapolated intercept value of *R*_α_(*m*_s_ = 0) (i.e., at zero sample mass) obtained from a regression of *R*_α_ as a function of sample mass *m*_s_ (as in Figs. 11 through 13). Intercept values of *R*_α_ obtained from regressions with other sample composition variables (e.g., *p*_aq_ or *N*_aq_ of Table 1—which also could be used as measures of changes in chemical quenching) were statistically equivalent to *R*_α_(*m*_s_ = 0) values. Six comparisons of *R*_α_(mean) to *R*_α_(*m*_s_ = 0), from the N, P, and Q sample series obtained with both LS counters, had differences ranging from −0.048 % to +0.10 %. The mean of the absolute values of the six differences was 0.037 % which may be taken as the component (c1) estimate of the relative standard uncertainty. The uncertainty on this uncertainty (reflected in the mean's sample (*n* = 6) standard deviation was ≈90 % on a relative basis. Alternatively, the above range of 0.148 % may be multiplied by a coefficient of 0.9 (for an unbiased estimate of a standard deviation from the range of two observations) to obtain a second estimate, call it (c2), of *u* ≈0.13 %. A third approach (c3) considers the difference (range) between the means of the most quenched and variable A and B sample series (after reduction by the known gravimetric dilution factor) and the mean *A*_α_ obtained from the *n* = 536 determinations data set (refer to Sec. 2.10.9 and Table 6). This difference yields, again after multiplying by 0.9, a *u* (c3) estimate of 0.20 %. This estimate includes the uncertainty in the gravimetric dilution factor (≃0.05 %). Lastly, for *u* (c4), one can consider the range in *A*_α_ obtained from the difference in the maximum mean *A*_α_ (with B series samples) to the minimum (Q series samples). There are either 9 or 18 mean values to consider in the range (see Table 6) depending on if one does or does not average the sample series results across both instruments. In either case, multiplying the range by a coefficient of ≈1/3 gives *u*(c4)≈0.12 %. On considering these values for *u*(c1), *u*(c2), *u* (c3), and *u*(c4) from the four approaches, a relative standard uncertainty of *u*(c)≈0.12 % seems reasonable. It also seems reasonable that this uncertainty component has in turn a relative uncertainty of ≈70 % (i.e., about 8 parts of 12 for the uncertainty on the uncertainty). The uncertainty in the LS detection efficiency must also be partially embodied in the uncertainty estimators for components (a) and (b) since the latter were obtained over a wide range of variables that might have influenced the efficiency.

Component (d) was estimated from the combination of comparisons between the quench indicating parameters *QIP* (either *H^#^* or *tSIE*) for the samples and matched blanks, and observed functional relationships between the blank counting rates (obtained over narrow time intervals) and the *QIP*. Data for the latter was obtained from a specially prepared series of 5 blanks having varying sample composition. The dependence of the blank counting rate *R*_b_ as a function of *QIP* was generally defined in terms of a linear regression having a fitted slope of d*R*_b_/d*QIP*. The background rate was for all intensive purposes independent of *QIP*. The fitted slopes were essentially zero with large inherent standard deviations: d*R*_b_/d*QIP* = 0.086 ± 0.026 for *H^#^* and 0.0077 ± 0.0086 for *tSIE*. For a difference Δ*QIP* between a sample *QIP* and that for its matched blank, the background counting rate difference Δ*R*_b_ due to the mismatch is Δ*R*_b_ = Δ*QIP*(d*R*_b_/d*QIP*). The relative standard uncertainty is then Δ*R*_b_/*R*_b_ and was typically a few tenths of a percent for Δ*QIP* differences of ± 1 unit of *H^#^* or 5 to 10 units of *tSIE*. The resulting contribution of component (d) to the uncertainty in *A*_α_, requires consideration of the sensitivity factor: e.g., *u*(d) = (*∂A*_α_/*∂R*_b_)(Δ*R*_b_/*R*_b_). The result, based on analyses of the P sample series on both LS counters was *u*(d) = 0.0039 % with a 60 % relative uncertainty on this uncertainty (i.e., for the 10 determinations of Δ*R*_b_/*R*_b_.

The variability in the background counting rates from measurement to measurement (i.e., component (e) uncertainty) was wholly embodied in the precision estimators for components (a) and (b).

The uncertainty due to cocktail stability over the measurement time in so far as it affected the chemical quenching and α detection efficiency (component (f) in Table 7) was estimated by taking the variability in the *QIP* (as given by the standard deviation *s_QIP_* determined from the multiple measurements of the *QIP* for a given sample) and multiplying it by the known change d*A*_α_/d*QIP* (or its equivalent d*R*_α_/d*QIP*) in *R*_α_ as a function of *QIP* for a given sample series. The estimator *s_QIP_* is a measure of the variability in *QIP* over the measurement time. For example, a linear regression to the *R*_α_ and *H^#^* data for the Q sample series had a fitted slope of d*R*_α_/d*H^#^* = 0.0057 ± 0.0029. For measurements of the same sample series, the relative standard deviation in *H^#^* for any sample ranged from 0.55 % to 0.80 %. The uncertainty in this case is then (d*R*_α_/d*H^#^*)*s_H#_*, which has corresponding values of 0.0015 % at a lower bound and 0.0068 % at an upper bound. Analyses of five cases for the N, P, and Q series with both spectrometers resulted in an overall relative standard uncertainty of *u*(f) = 0.0048 %. Again, as for the component (c) and (d) cases, the uncertainty in this uncertainty estimate is large, approaching 75 %. The relative uncertainty in *QIP* was *s_QIP_* = 0.3 % to 0.9 %. Values of d*R_α_*/d*QIP* ranged from 0.0001 to 0.006 with accompanying relative uncertainties in d*R_α_*/d*QIP* ranging from 500 % to 50 %. One must further note that some part of the random variability in the cocktail stability must be included in all of the replicates making up the component (a) and (b) estimators.

The LS detection efficiency varies slightly with sample volume and is at an optimal maximum (in these sample configurations) for volumes somewhere between 8 mL and 16 mL. The component (g) standard uncertainty estimate was obtained from comparing the differences in the mean *A*_α_ values obtained with samples having *m*_tot_≃10 g to 11 g and with that for samples having *m*_tot_≃14 g to 15 g. Part of the variability in LS efficiency due to sample volume must also be embodied in components (a) and (b).

Other LS counting interferences (such as those arising from discharges of static electricity on the LS sample vials or in the LS counter; wall effects due to solvent diffusion into plastic vials; sample heterogeneity which results in differing efficiency in the various heterogeneous phases or components; and random electronic noise) are all sporadic in nature. These effects would increase the random variability in the measurement results. Considering the wide range of variables over which the measurements were obtained (with different types of LS vials, over widely varying sample composition, with different instruments and operating parameters, and at many different times over more than one year), the uncertainty in the LS efficiency due to these sporadically occurring interfering effects are considered to be wholly incorporated within the component (a) and (b) uncertainty estimators.

For component (i), the uncertainty in live time determinations was estimated by assuming that the deadtime corrections that are automatically performed by the LS counters are appropriately made to within ± 10 % (which is a reasonable assumption given the state of electronics for modern ADC-based counting instruments [29]) and on consideration of the counting rates of the LS samples and the known resolving times for the LS instruments (further assuming that the deadtime intervals are fixed). The relative uncertainties calculated on this basis were 0.01 % and 0.03 % in worst cases for the Packard and Beckman instruments, respectively. Given that roughly half of the total number of measurements of *R*_α_ were performed with each instrument, the relative standard uncertainty *u* (i) was taken to be 0.02 %.

The variability in LS sample mass measurements (component (j) uncertainty) is wholly embodied in the uncertainty estimators for components (a) and (b) in as much as the mean *R*_α_ was based on 27 independent sample masses (with each mass determination having an independent confirmation — see Sec. 2.2.3).

For component (k), the estimated relative standard uncertainty in the gravimetric determination for any one LS sample is 0.05 % and was treated *in extenso* in Sec. 2.2.3.

The relative standard uncertainties in the two corrections *k*_ZE_ and *k*_EC_c (for the (1) and (m) components) were respectively addressed in Sec. 2.10.2 and Appendix A.

Timing uncertainties were negligible considering the long half-lives involved in these measurements. For a timing error of 1 min (which is almost inconceivable), the relative uncertainty in the ^209^Po decay correction is less than 1.3×10^−6^ %. Also, any timing variabilities would certainly have been averaged out over the 536 measurements contained in components (a) and (b).

For components (o), (p), and (q), the uncertainties in the corrections for ^209^Po decay and the ^208^Po impurity (with its respective decay) were derived assuming relative uncertainties of *u*(λ^209^Pο) = 4.9 %, *u*(λ^208^Pο) = 0.069 %, and *u*(*I*) = 8.1 %. The uncertainties in the decay constants λ were obtained from the Evaluated Nuclear Structure Data File [30] while that for the ^208^Po to ^209^Po α-emission rate impurity ratio ·I was given in Sec. 2.9. The mean *R*_α_ was obtained from determinations separated by more than a year (with all determinations decay and impurity corrected to an identical reference time). Hence, the magnitude of the corrections (and hence their uncertainties) are quite disparate. The 1993 LS measurement results were corrected for decay times from 0.7 yr to just over 1 yr whereas those obtained in 1994 were at most only corrected over times of a few weeks (<0.04 yr). One may therefore consider the ranges of uncertainty values over these decay times. For decay times ranging from *T*≃0.04 yr to ≃1 yr (see equations in Sec. 2.4.1), the propagated relative uncertainty in the ^209^Po decay correction ranges from 0.0013 % to 0.033 %. A mid-value of *u*(o)≃0.02 % may thus be used as an estimate of the standard uncertainty for this component. Similarly, the correction for the ^208^Po impurity over this same time interval ranges from about *u*(p)≃0.01 % to 0.008 %. The uncertainty contribution from ^208^Po decay is negligible with an estimated relative uncertainty component of *u*(q)≃8×10^−7^ % for *T*≃0.04 yr to <2×10^−5^ % for *T*≃1 yr. The inherent random variabilities in these three corrections are also of necessity partially embodied in the uncertainty estimators for components (a) and (b).

The treatment used to propagate these components into a combined standard uncertainty and an “overall” expanded uncertainty was outlined in Sec. 2.1.4. One should also recall that the data set of 536 *R*_α_ values that largely comprise these uncertainties was shown to be normally distributed (Sec. 2.10.2).

## 3. Concluding Notes

The prepared and calibrated ^209^Po solution standard will be certified and disseminated as NIST SRM 4326 with the following specifications:

Form: Solution of nominal 2 mol · L^−1^ HCl in a flame-sealed NIST 5 mL borosilicate glass ampoule
Solution mass: (5.1597 ± 0.0024) g
Solution density: (1.031 ± 0.004) g · mL^−1^ at 22 °C
Alpha emission rate concentration: (85.42 ±0.29) s^−1^ · g^−1^
Known radionuclidic (α emission) impurities: (0.106 ± 0.017) s^−1^ · g^−1^ from ^208^Po
Reference time: 1200 EST 15 March 1994

The calibration was based on 4πα LS spectrometry using two LS measurement systems, with confirmatory measurements by 2πα gas-flow proportional counting. The mean α emission rate concentration was derived from 536 independent determinations of the LS counting rate, using 27 counting sources (of widely varying chemical quenching and sample composition) that were in turn obtained from 7 randomly selected sealed ampoules of the ^209^Po solution standard. The determinations were performed over a long (≃1 yr) time interval. Photon-and alpha-emission impurity analyses were conducted by γ-ray spectrometry with two intrinsic Ge detectors for the former, and by α spectrometry with a Si surface-barrier junction detector for the latter.

The 4πα LS-measurement calibration of ^209^Po (a nearly pure α emitter) would have been routinely straightforward otherwise, but was confounded by the existence of a low-energy level in ^205^Pb (fed by the α decay of ^209^Po) that was previously unknown to be a long-lived isomeric state. This finding (or more accurately, its converse) required the exhaustive LS spectral investigations that were performed as well as the large number of LS measurements that were conducted with large variations in chemical quenching and sample composition conditions.

We may ultimately conclude by noting that this work is reported here in such depth and exacting detail for two reasons. Firstly, because of the complexity of this calibration; and secondly, and perhaps more importantly, to provide a documented and archival record of some of the radionuclidic metrology procedures routinely practiced by the NIST Radioactivity Group.

## Figures and Tables

**Table 1:** LS sample compositions

Sample Identities	^209^Po Solution^a^	*m*_s_ Range^b^ (g)	*m*_tot_ Range^c^ (g)	*p*_aq_ Range^d^ (%)	*N*_aq_ Range^c^ (mol · L^−1^)
A1–A2	A	0.05–0.07	10.10–10.08	0.54–0.65	2.00
B1–B3	B	0.05–0.06	10.09–10.10	0.52–0.55	2.00
B4–B6	B	0.05	10.09–11.14	9.44–9.84	0.097–0.101
B4x–B6x	B	0.05	11.35–11.42	11.5–12.1	1.98–2.08
MA1–MA3	M (amp. 39)	1.01–1.04	11.05–11.08	9.11–9.34	2.00
MB1–MB3	M (amp. 93)	0.99–1.04	11.03–11.08	8.97–9.36	2.00
MC1–MC3	M (amp. 129)	0.99–1.02	11.03–11.06	8.97–9.23	2.00
MD1–MD3	M (amp. 205)	1.00–1.01	11.04–11.05	9.08–9.15	2.00
N1–N6	M (amp. 47)	0.12–1.01	11.11–11.13	9.03–9.21	0.225–2.00
P1–P5	M (amp. 40)	0.11–0.97	10.98–11.03	8.87–9.15	0.209–2.00
Q1–Q4	M (amp. 29)	0.05–0.71	10.06–11.03	0.534–9.24	0.225–2.00
Q5–Q8	M (amp. 29)	0.13–1.00	14.11–14.99	0.892–6.70	2.00

a See Fig. 1.

b Approximate mass of ^209^Po solution used in preparing the sample.

c Approximate total mass of sample composed of the sum of *m*_s_, *m*_r_, *m*_w_, and *m*_a_. Refer to text.

d Approximate water content of the sample on a mass percentage basis.

e Approximate acid content (normality) in the sample aqueous phase.

**Table 2:** LS measurement results for the M sample series obtained with the Beckman counting system

Sample identity	Number of samples	*n*^a^	*R*_α_ mean^b^ (s^−1^ · g^−1^)	*s*^c^ (s^−1^ · g^−1^)	*s*_m_^d^ (%)
Replicate measurements on single samples					
MA1	1	10	85.414	0.277	0.103
MA2	1	10	85.452	0.222	0.082
MA3	1	10	85.617	0.275	0.102
MB1	1	10	85.554	0.347	0.128
MB2	1	10	85.437	0.243	0.090
MB3	1	10	85.353	0.125	0.046
MC1	1	10	85.370	0.264	0.098
MC2	1	10	85.348	0.251	0.093
MC3	1	10	85.282	0.169	0.063
MD1	1	10	85.431	0.164	0.061
MD2	1	10	85.416	0.258	0.096
MD3	1	10	85.545	0.224	0.083

Replicate measurements on samples from a single ampoule					
MA1–MA3	3	30	85.494	0.266	0.057
MB1–MB3	3	30	85.448	0.260	0.056
MC1–MC3	3	30	85.333	0.227	0.049
MD1–MD3	3	30	85.464	0.219	0.047

All measurements					
MA1–MD3	12	120	85.435	0.253	0.027

a Total number of measurements on all samples in the series.

b Mean of the ^209^Po α counting rate concentration, with ^208^Po impurity and decay corrections to the reference time 1200 EST 15 March 1994 (see Sec. 2.4.1).

c Standard deviation for the *n* determinations.

d Relative standard deviation of the mean in percent for the *n* determinations.

**Table 3:** LS measurement results for the M sample series obtained with the Packard counting system

Sample identity	Number of samples	*n*^a^	*R*_α_ mean^b^ (s^−1^ · g^−1^)	*s*^c^ (s^−1^ · g^−1^)	*s*_m_^d^ (%)
Replicate measurements on single samples					
MA1	1	10	85.563	0.197	0.073
MA2	1	10	85.370	0.190	0.070
MA3	1	10	85.321	0.139	0.052
MB1	1	10	85.446	0.241	0.089
MB2	1	10	85.459	0.260	0.096
MB3	1	10	85.400	0.229	0.085
MC1	1	10	85.304	0.200	0.074
MC2	1	10	85.447	0.317	0.117
MC3	1	10	85.562	0.275	0.102
MD1	1	10	85.394	0.215	0.080
MD2	1	10	85.445	0.202	0.075
MD3	1	10	85.478	0.233	0.086

Replicate measurements on samples from a single ampoule					
MA1–MA3	3	30	85.418	0.201	0.043
MB1–MB3	3	30	85.435	0.237	0.051
MC1–MC3	3	30	85.438	0.280	0.043
MD1–MD3	3	30	85.439	0.212	0.045

All measurements					
MA1–MD3	12	120	85.433	0.232	0.025

a Total number of measurements on all samples in the series.

b Mean of the ^209^Po α counting rate concentration, with ^208^Po impurity and decay corrections to the reference time 1200 EST 15 March 1994 (see Sec. 2.4.1).

c Standard deviation for the *n* determinations.

d Relative standard deviation of the mean in percent for the *n* determinations.

**Table 4:** LS measurement results for the P and Q sample series obtained with the Beckman and Packard counting systems

	Beckman				Packard			
	Samples P1–P5		Samples Q1–Q4		Samples P1–P5		Samples Q5–Q8	
Parameter	*R*_αe_^a^	*R*_α_^b^	*R*_αe_^a^	*R*_α_^b^	*R*_αe_^a^	*R*_α_^b^	*R*_αe_^a^	*R*_αe_^b^
*n*_s_^c^	5	5	4	4	5	5	4	4
*n*_m_^d^	62	62	48	48	25	25	71	71
Mean *R*^e^ (s^−1^ · g^−1^)	87.787	85.532	88.011	85.460	89.278	85.553	89.847	85.528
*s*^f^ (s^−1^ · g^−1^)	0.226	0.175	0.512	0.310	0.323	0.264	0.540	0.184
*s*_m_^g^ (%)	0.033	0.026	0.084	0.052	0.072	0.062	0.071	0.026
Intercept *R*^h^ (s^−1^ · g^−1^)	87.967	85.553	88.301	85.374	89.508	85.536	90.643	85.518
*s*_f_^i^ (s^−1^ · g^−1^)	0.059	0.040	0.254	0.057	0.047	0.042	0.117	0.043

a Counting rate concentration obtained from integrating the full-energy LS spectra (see Sec. 2.4.1), with ^208^Po and decay corrections to the reference time 1200 EST 15 March 1994.

b ^209^Po α counting rate concentration (see Sec. 2.4.1), with ^208^Po and decay corrections to the reference time 1200 EST 15 March 1994.

c Number of samples in the sample series.

d Total number of measurements on all samples in the series.

e Mean counting rate concentration.

f Standard deviation in the counting rate concentration for the *n* determinations; *v* = *(n*_m_−1) degrees of freedom.

g Relative standard deviation of the mean in percent for the *n* determinations; $s_{m}=100\;s/m\;\sqrt{n_{m}}$.

h Fitted intercept value (at *m*_s_ = 0) for the counting rate concentration from unweighted linear regressions on the mean *R* for each of the *n*_s_ samples in the series (see Fig. 13).

i Standard deviation for the fitted intercept value; *v* = (*n*_s_−2) degrees of freedom.

**Table 5:** Determinations of the ^208^Po/^209^Po impurity ratio by α spectrometry

Source identity	Counting time (s)	^209^Po 4.88 MeV α^a^ (s^−1^ · g^−1^)	^208^Po 5.11 MeV α^a^ (s^−1^ · g^−1^)	*s*_p_^b^ %	*s*_b_^c^ %	^208^Po/^09^Po^a^ Impurity ratio
LX2	3.5×10^5^	22.40	0.0256	3.9	2.1	0.001144
LX2	6.0×10^5^	22.07	0.0297	2.8	1.6	0.001344
LX1	6.0×10^5^	21.46	0.0265	1.6	2.0	0.001236

a As of the reference time 1200 EST 15 March 1994. The counting rate concentrations for the ^209^Po and ^208^Po peaks are in traditional units of “counts per second per gram.”

b Relative standard deviation in percent for the assumed Poisson-distributed total statistical “counting error” which is given by $s_{p}100=\sqrt{(N_{\alpha 1}+N_{b1})/{\left(N_{\alpha 1}-N_{b1}\right)}^{2}+(N_{\alpha 2}+N_{b2})/{\left(N_{\alpha 2}-N_{b2}\right)}^{2}}$ where *N*_α1_ and *N*_α2_ are the total gross counts in the two α peaks and *N*_b1_ and *N*_b2_ are the estimated total counts in the two underlying and extrapolated peak backgrounds.

c Relative standard uncertainty in percent for locating and determining the two underlying α peak backgrounds (propagated from estimated uncertainties on each peak).

**Table 6:** Summary of all LS measurement results for mean *R*_α_ obtained with Beckman and Packard counting systems

			Results with Beckman			Results with Packard		
Sample identities	^209^Po Solution	Number of samples	*n*^a^	*R*_α_ mean^b^ (s^−1^ · g^−1^)	*s*_m_ %^c^	*n*^a^	*R*_α_ mean^b^ (s^−1^ · g^−1^)	*s*_m_ %^c^
A1–A2	A	2	59	12566	0.016	34	12557	0.019
B1–Bbx	B	9	138	12557	0.020	115	12552	0.021
all A & B	A/B	11	197	12560	0.015	149	12553	0.017
MA1–MA3	M (amp. 39)	3	30	85.494	0.057	30	85.418	0.043
MB1–MB3	M (amp. 93)	3	30	85.448	0.056	30	85.435	0.051
MC1–MC3	M (amp. 129)	3	30	85.333	0.049	30	85.438	0.043
MD1–MD3	M (amp. 205)	3	30	85.464	0.047	30	85.439	0.045
N1–N6	M (amp. 47)	6	30	85.492	0.045	60	85.424	0.019
all M & N	M	18	150	85.446	0.023	180	85.430	0.018
P1–P5	M (amp. 40)	5	62	85.532	0.026	25	85.553	0.062
Q1–Q4	M (amp. 29)	4	48	85.460	0.052			
Q5–Q8	M (amp. 29)	4				71	85.528	0.026
all P & Q	M	9	110	85.501	0.027	96	85.535	0.025
All M–Q	M	27	260	85.469	0.018	276	85.466	0.015

a Total number of measurements on all samples in the series.

b Mean of the ^209^To α counting rate concentration, with ^208^Po impurity and decay corrections to the reference time 1200 EST 15 March 1994 (see Sec. 2.4.1).

c Relative standard deviation of the mean in percent for the *n* determinations.

**Table 7:** Summary of the uncertainty analyses for the ^209^Po α emission rate concentration

Uncertainty components and propagated uncertainties^a^	Relative uncertainties %
(a) LS measurement reproducibility	0.012
(b) Variability in ^209^Po α emission rate concentration for any ampoule of the SRM	0.056
(c) LS detection efficiency (system and chemical quenching (correction) variability)	0.12 (and PE)^b^
(d) Variability in matching LS samples and blanks for background subtractions	0.004
(e) Background measurement imprecision	WE^b^
(f) LS cocktail stability over measurement time (quench variability)	0.005 (and PE)^b^
(g) LS sample volume effect on LS efficiency	0.03 (and PE)^b^
(h) Other LS counting interferences (e.g., wall effects, sample heterogeneity, static electricity, etc.)	WE^b^
(i) Live time determinations for LS counting time intervals	0.02
(j) Variability in LS sample masses	WE^b^
(k) Gravimetric (mass) determination for any one LS sample	0.05
(l) Extrapolation of LS count rate to zero energy and correction for subtraction of 2.3 keV ce response	0.06
(m) Correction of LS response for EC branch decay	0.06
(n) Timing	negligible (and PE)^b^
(o) Decay corrections for ^209^Po from measurement time to common reference time	0.02 (and PE)^b^
(p) ^208^Po impurity correction for embodied uncertainty in ^208^Po/^209^Po impurity ratio	0.009 (and PE)^b^
(q) Decay corrections for ^208^Po from measurement time to common reference time	negligible (and PE)^b^
Combined standard uncertainty,^a^ *u*	0.17
Expanded uncertainty^a^ for *k* = 2, *U = ku*	0.34

a See accompanying text and refer to Sec. 2.1.4.

b The relative uncertainty for this component is wholly (WE), or in part (PE), embodied in the relative standard uncertainties of components (a) and (b).

## Footnotes

1 Historical note: The use of LS counting as an “absolute” a standardization method was first proposed by Basson and Steyn [7] in 1954. Shortly thereafter, Basson [8] and Horrocks and Studier [9] demonstrated the feasibility and applicability of 4πα LS spectrometry for absolute standardization. Its use has since then become a fairly routine direct measurement methodology for a emitting radionuclides, including within the NIST Radioactivity Group.

2 Certain commercial equipment, instruments, or materials are identified in this paper to foster understanding. Such identification does not imply recommendation or endorsement by the National Institute of Standards and Technology, nor does it imply that the materials or equipment identified are necessarily the best available for the purpose.

3 Because all of the counting rate concentrations that follow, such as for *R*_α_ *R*_αe_, and *R*_e_, include the concept of “counts” in their definitions, their proper units are s^−1^ · g^−1^ rather than the traditionally used “counts per second per gram.”

4 The total “counting error” devolves from the assumption that the radioactive decay process is Poisson distributed and that its variance (*N*) is equal to the mean number of total observed counts (*N*). The propagated *s*_p_ is expressed as a relative standard deviation and is merely given by $\sqrt{N_{S}+N_{B}}/(N_{S}-N_{B})$ where *N*_S_ is the total number of counts obtained in measuring the sample and *N*_B_ is the respective total number of background counts, and where *N*_s_ and *N*_B_ are obtained over counting time intervals of equal duration. The standard deviation in the counting rate concentration *R* (due to the counting errors) is then the product *s*_p_(*R*) =*s*_p_*R*.

5 Other explanations can not be definitively excluded. In a private communication, Dodson [17] reported that he has only observed this type of doublet effect with ^241^Am, and not with other a emitters like ^226^Ra or ^238^Pu. He is lead to believe it may be a sample preparation effect since he never observed it with samples prepared by extractions (i.e., with extractive scintillators). In a set of quenched ^241^Am “standards”, a doublet was “not initially present, but later the doublet appeared at the same time that the emulsifier precipitated, similar to a two-phase condition” [17]. This explanation, however, is not compelling for the present situation. Firstly, the observed doublets in the ^209^Po spectra appear to be composed of two symmetric components of nearly equal magnitude. It is hard to imagine that the polonium was in different chemical states or phases to nearly exactly equal extents. Secondly, the observed doublets were most pronounced in the least aged and least quenched samples, and not the converse. Although the “doublets” were observed in spectra obtained with both spectrometers, their appearance was invariably more pronounced (or defined) in those obtained with the Packard instrument.

6 In correct usage, alpha emission rate concentrations have units of s^−1^ · g^−1^, rather than the traditional units of “alphas per second per gram.”

7 These emission rate concentrations have traditional units of “photons per second per gram.”

8 Several standard statistical tests were performed to test the data set for randomness and for its distributional form. These included both graphical evaluations and calculations of various distributional measures. The 536-value data set appeared very normally distributed on a normal probability plot, particularly if the two largest values (see Fig. 27) in the set were excluded. Tests for normality using a probability plot correlation coefficient test gave coefficients of *r* = 0.998 for the slightly smaller *n* = 534 data set and *r* = 0.996 for the entire (*n* = 536) data set.

## 4. Appendix A. Correction for LS Response to ^209^Po Electron Capture Decay

The LS responses from the counting sources of the ^209^Po standard solution included contributions from some portion of radiations arising from the ^209^Po EC decay mode. Conversion of the previously given mean counting rate concentration *R*_α_ to a ^209^Po α-emission rate concentration requires a correction for these EC-contributing LS responses. The correction can be approximated by consideration of the known ^209^Po decay and atomic and nuclear structure parameters, and estimation of LS detection efficiencies. The following notation will be used:

*I*_α_: ^209^Po α decay intensity; number of emitted α particles per decay of ^209^Po
*I*_EC_: ^209^Po EC decay intensity; total number of captures per decay of ^209^Po
*I_j_*: intensity of the *j*^th^ radiation component (electrons or photons) arising from the EC decay of ^209^Po
*ϵ_j_*: LS detection efficiency for the *j*^th^ component
*P*_K_: K-shell EC probability; number of K-shell electron captures (holes) per EC decay
*ω*_κ_: K-shell fluorescence yield; number of K x rays emitted per K-shell hole
*P*_L_,*P*_M_,*P*_N_,…: EC probability for L-shell capture, M-shell capture, N-shell …., respectively; number of electron captures (holes) in the respective shells per EC decay
*B*_K_,B_L_,B_M_,…: atomic binding energy for the K shell, L shell, M shell, …. electrons
${\bar{\omega }}_{L}$: average L-shell fluorescence yield for all three L subshells (including x rays following Coster-Kronig subshell rearrangements); total number of L x rays emitted per initial L-shell hole
${\bar{\omega }}_{M}$: average M-shell fluorescence yield for all five M subshells; total number of M x rays emitted per initial M-shell hole
α: total internal conversion coefficient for a γ transition; ratio of the number of de-excitations which occur by conversion (with the emission of an atomic electron) to the number of γ-ray photons emitted.

The LS counting rate concentration *R*_α_, when normalized to the ^209^Po activity concentration, is given by *I*_α_ +*fI*_EC_ where *f* is the fraction of all radiations from the EC branch that were detected. The correction factor *k*_EC_ is then

$$k_{\mathrm{EC}}=I_{\alpha }/(I_{\alpha }+fI_{\mathrm{EC}})=1/[1+f(I_{\mathrm{EC}}/I_{\alpha })].$$

The ratio *I*_EC_/*I*_α_, from the critical evaluation of Martin [11], is equal to (0.0048 ± 0.0004)/(0.9952 ± 0.0004) = 0.0048 ± 0.0004. The quantity *f* is a summation *f* = ∑*ϵ_j_I_j_/I*_EC_ over all *j* photons and electrons arising from the EC decay branch, and where *ϵ_j_* and *I_j_/I*_EC_ are the LS detection efficiency and relative intensity for the *j*^th^ component. A lower limit on the magnitude of *k*_EC_ may be established. If all *j* components of the EC branch are detected with 100 % efficiency (i.e., *f* = 1), then *k*_EC_ = 0.9952 ± 0.0004 is the lower limit. The upper limit, of course, would be *k*_EC_ = 1 with *f* = 0 (i.e., none of the EC radiations are detected).

Electron capture decay of ^209^Po is a second forbidden unique EC transition (Δ*J*^π^ = 1/2^−^→7/2^−^). The result is that K-shell capture is relatively low, and that significant EC contributions occur from higher atomic shells. This in turn results in the emission of lower energy x rays (L, M, … instead of K x rays) and greater Auger electron intensities (since *ῶ*_L_, *ῶ*_M_, … < ω_K_). LS detection efficiencies for these lower energy x rays and Auger electrons are larger that that for K x rays which are usually the predominant emissions in high atomic number EC transitions.

The relative intensity of K x rays is *I*_Kx_/*I*_EC_ = P_κωκ_. The capture ratio *P*_L+M+N+_*_…_/P*_K_ was experimentally determined by Mandel et al. [13] to be 0.42 ± 0.07, and which may be compared to a value of 0.39 from theoretical estimates of Behrens and Buhring [31]. If one adopts a value of 0.4 ± 0.1 (and given that *P*_K_*+P*_L_*+P*_M_*+… = 1*), then *P*_K_ = 0.70±0.015. With ωκ for Bi taken as = 0.970 ± 0.015 [32,33], *I*_Kx_/*I*_EC_ = 0.68 ±0.05. The LS detection efficiency for these rather energetic 74.8 keV to 89.8 keV x rays is small. Photoelectric absorption is negligible. Their interaction in the LS cocktail is primarily by Compton scattering which results in Compton electrons from zero energy to about 10 keV. As a result, the efficiency is estimated to be from near zero to perhaps 10 % to 20 %. It will be assumed that 5 % of the K x rays are detected, but the upper uncertainty limit on the detection efficiency will be taken as 15 % such that *ϵ*_Kx_*I*_Kx_/*I*_EC_ = (0.68 ± 0.05) $(0.05_{-0.05}^{+0.10})=0.34_{-0.034}^{+0.10}.$ The relative intensity of K-shell Auger electrons is *I*_Ke_*/I*_EC_*=P*_K_(1 − ω_K_) = 0.021 ± 0.002. They have discrete energies ranging from about 58 keV to 90 keV given by the atomic binding energy differences $\left(B_{K}-2B_{L_{I}}\right)$, $\left(B_{K}-2B_{L_{\mathrm{II}}}\right)$, $\left(B_{K}-2B_{L_{\mathrm{III}}}\right)$, $\left(B_{K}-2B_{M_{I}}\right)$, *…* $\left(B_{K}-2B_{M_{V}}\right)$, $\left(B_{K}-2B_{N_{I}}\right)$, … etc., and are assumed to be detected with 100 % efficiency. Therefore, *ϵ*_Ke_*I*_Ke_/*I*_EC_ = 0.021 ± 0.002.

The x rays and Auger electrons for L-shell capture can be treated analogously. The relative intensity of L x rays is *I*_Lx_/*I*_EC_ = (*P*_L_*/P*_K_)*P*_K_*ῶ*_L_. A capture ratio of *P*_L_/*P*_K_ = 0.3 ± 0.1 is taken from the theoretical values $P_{L_{I+\mathrm{II}}}/P_{K}=0.28$ and $P_{L_{\mathrm{III}}}/P_{K}=0.02$ [31]. The average fluorescence yield for all three L sub-shells is *ῶ*_L_ = 0.40 ± 0.06 which was obtained from the mean of six experimental determinations tabulated by Bambynek et al. [32]. Hubbell et al. [33] in a very recent review reported a value of *ῶ*_L_ = 0.382. The L x rays have energies of around 11 keV to 15 keV, and have a range of about 0.6 cm in the LS cocktails which is about comparable to the average geometric path length in the scintillator volume. They are assumed to be detected with an efficiency of *ϵ*_Lx_ = 0.5 ± 0.25, so that *ϵ*_Lx_(*I*_Lx_/*I*_EC_) = 0.042 ± 0.026. The L Auger electrons, having energies of around 5.4 keV to 16 keV, are assumed to be detected with 100 % efficiency as for the K Auger electrons. The detected fraction of their relative intensity is thus *ϵ*_Le_(*I*_Le_/*I*_EC_) = *ϵ*_Le_(*P*_L_*/P*_K_)*P*_K_(1−*ῶ*_L_) = 0.126 ± 0.048.

Higher atomic shell (M + N + …) EC events have considerably lower relative capture probabilities. A value of *P*_M+N+…_/*P*_K_ = 0.08±0.01 is estimated, again from theoretical predictions: $P_{M_{I+\mathrm{II}}}/P_{K}=0.07$; $P_{M_{\mathrm{III}}}/P_{K}=5.639\times 10-3$; $P_{M_{\mathrm{IV}}}/P_{K}=6.19\times 10-5$ [31]. The relative intensities for the x rays are relatively low and are ignored. Given *ῶ*_M_ = 0.035 ± 0.005 [32,33], *I*_Mx_*/I*_EC_≃0.001 for example. The M shell Auger electrons range in energy from 0.7 keV to about 3.9 keV. All N shell Auger electrons have energies of less than 1 keV. For convenience (and in the absence of additional information with which to make better approximations), we can merely assume that all of these higher shell Auger electrons are collectively detected with 50 % efficiency and with a somewhat arbitrarily assigned relative uncertainty of ±50 % on the efficiency. With all Auger electron yields $\left(1-{\bar{\omega }}_{M}\right),\left(1-{\bar{\omega }}_{N}\right),$ …, etc. taken to be equal to unity, the detected fraction of the relative intensity is therefore assumed to be *∈*_M+N+…_(*I*_Me+Ne+…_/*I*_EC_)=0.028±0.014.

Detection of the 896.6 keV γ ray and its attendant conversion electron (see Fig. 2) is neglected. Firstly, these are in prompt coincidence with the radiative and non-radiative EC decays, and therefore would only be detected by the LS spectrometers if and when the x rays and Auger electrons were not detected. Secondly, the LS detection efficiency for the γ ray is virtually nil, and the relative intensity for the conversion electron, given by the transitions total conversion coefficient *α*_T_, is very small. Using *I*_γ_ = 0.0047 ±0.0004 and α_γ_ = 0.0209 ± 0.0005 from Martin [11], the relative intensity of the conversion electron is *I_cc_/I*_EC_=0.020 ± 0.002; and, as stated, it would only be detected for that fraction of the EC events (primarily x rays) that are not detected. The undetected fraction of x rays and Auger electrons (based on summing all of the detected relative intensity contributions given above) is roughly 75 %. Therefore, the detected relative intensity for the conversion electron would be $\tilde{<}0.015$ as a maximum.

The correction factor may thus be approximated by summation of

$$\begin{array}{c} f=\in_{\mathrm{Kx}}\left(\frac{I_{\mathrm{Kx}}}{I_{\mathrm{EC}}}\right)+\in_{\mathrm{Ke}}\left(\frac{I_{\mathrm{Ke}}}{I_{\mathrm{EC}}}\right)+\in_{\mathrm{Ix}}\left(\frac{I_{\mathrm{Lx}}}{I_{\mathrm{EC}}}\right)+e_{Le}\left(\frac{I_{\mathrm{Le}}}{I_{\mathrm{EC}}}\right)\\ +\in_{\mathrm{Me}+\mathrm{Ne}+\ldots }\left(\frac{I_{\mathrm{Me}+\mathrm{Ne}+\ldots }}{I_{\mathrm{Ec}}}\right)=0.251_{+0.080}^{-0.117} \end{array}$$

(or ƒ=0.266 if the maximum contribution from the conversion electron is included); which results in $k_{\mathrm{Ec}}=0.9988_{-0.0004}^{+0.0006}$ The relative standard uncertainty on *k*_EC_ will be taken as the larger upper bound, i.e., *u* = ±0.06 %.

### Note Added in Proof

Standard Reference Material SRM 4326 has been issued with a relative expanded uncertainty of 0.42 % for the ^209^Po alpha emission rate concentration. This is slightly revised from that given here (0.34 %). The difference is mainly due to more conservative estimates in the livetime measurement and LS detection efficiency uncertainty components, and in the treatment of the component uncertainties for possible undetected alpha-particle-emitting and photon-emitting impurities at their limits of detection. Considering the large “uncertainty in the uncertainty” associated with these estimates, the two values may be viewed as essentially equivalent.
